# Regulatory Mechanisms of Metamorphic Neuronal Remodeling Revealed Through a Genome-Wide Modifier Screen in *Drosophila melanogaster*

**DOI:** 10.1534/genetics.117.200378

**Published:** 2017-05-05

**Authors:** Dahong Chen, Tingting Gu, Tom N. Pham, Montgomery J. Zachary, Randall S. Hewes

**Affiliations:** Department of Biology, University of Oklahoma, Norman, Oklahoma 73019

**Keywords:** *shep*, neuronal remodeling, metamorphosis, peptidergic neurons, BMP signaling

## Abstract

During development, neuronal remodeling shapes neuronal connections to establish fully mature and functional nervous systems. Our previous studies have shown that the RNA-binding factor *alan shepard* (*shep*) is an important regulator of neuronal remodeling during metamorphosis in *Drosophila melanogaster*, and loss of *shep* leads to smaller soma size and fewer neurites in a stage-dependent manner. To shed light on the mechanisms by which *shep* regulates neuronal remodeling, we conducted a genetic modifier screen for suppressors of *shep*-dependent wing expansion defects and cellular morphological defects in a set of peptidergic neurons, the bursicon neurons, that promote posteclosion wing expansion. Out of 702 screened deficiencies that covered 86% of euchromatic genes, we isolated 24 deficiencies as candidate suppressors, and 12 of them at least partially suppressed morphological defects in *shep* mutant bursicon neurons. With RNA interference and mutant alleles of individual genes, we identified *Daughters against dpp* (*Dad*) and *Olig family* (*Oli*) as *shep* suppressor genes, and both of them restored the adult cellular morphology of *shep*-depleted bursicon neurons. *Dad* encodes an inhibitory Smad protein that inhibits bone morphogenetic protein (BMP) signaling, raising the possibility that *shep* interacted with BMP signaling through antagonism of *Dad*. By manipulating expression of the BMP receptor *tkv*, we found that activated BMP signaling was sufficient to rescue loss-of-*shep* phenotypes. These findings reveal mechanisms of *shep* regulation during neuronal development, and they highlight a novel genetic *shep* interaction with the BMP signaling pathway that controls morphogenesis in mature, terminally differentiated neurons during metamorphosis.

NEURONAL remodeling is a critical process that nervous systems undergo during development to become fully mature and functional. This process has been characterized and investigated in a wide range of organisms including worms, insects, mice, and humans (Zheng *et al.* 2003; Gogtay *et al.* 2004; Dunn and Wong 2012; Thompson-Peer *et al.* 2012). Studies over the past few decades have suggested that dysregulated neuronal remodeling leads to abnormal neuronal organization and may contribute to neurological diseases such as schizophrenia (Feinberg 1982; Faludi and Mirnics 2011; Sekar *et al.* 2016). *Drosophila melanogaster* is one of the best model organisms to study neuronal remodeling because of the dramatic structural and functional reorganization of its nervous system during metamorphosis (Weeks 2003; Williams and Truman 2005b). In addition to the programmed cell death of larval neurons and birth of adult neurons during this process, numerous larval neurons persist through metamorphosis. The persistent neurons undergo precisely regulated remodeling involving pruning of larval neurites and outgrowth of adult neurites. Well-characterized examples of neuronal remodeling include the mushroom body γ-neurons (Zheng *et al.* 2003; Awasaki and Lee 2011; Yu *et al.* 2013), thoracic ventral Tv4 neurons (Schubiger *et al.* 1998, 2003; Brown *et al.* 2006), peripheral sensory Da neurons (Kuo *et al.* 2005; Williams and Truman 2005a), and bursicon neurons (Zhao *et al.* 2008). In multiple cell types, TGFβ signaling and the nuclear receptors Ftz-f1 and Hr39 have been shown to regulate neurite pruning by promoting EcR-B1 expression specifically in remodeling neurons (Schubiger *et al.* 1998; Zheng *et al.* 2003; Williams and Truman 2005a; Brown *et al.* 2006; Liu *et al.* 2010; Awasaki and Lee 2011; Boulanger *et al.* 2012). Some downstream effectors of EcR-B1 in the pruning process have also been identified (Hoopfer *et al.* 2008; Kirilly *et al.* 2009). In contrast, while a few studies have begun to shed light on the outgrowth phase of the remodeling process (Jefferis *et al.* 2004; Yaniv *et al.* 2012), the mechanisms governing outgrowth largely remain a mystery.

The *alan shepard* (*shep*) gene is widely and primarily expressed in the nervous system and has been shown to regulate metamorphic neuronal outgrowth and development (Chen *et al.* 2014; Schachtner *et al.* 2015). Loss of *shep* leads to defective outgrowth of peptidergic bursicon neurons, developmental lethality, and behavioral defects, all of which are largely adult-specific (Chen *et al.* 2014). Loss of *shep* also interferes with the development of nociceptive and proprioceptive neurons in the larval peripheral nervous system (Schachtner *et al.* 2015). In addition, the *shep* gene has been identified in a number of screens for factors involved in gravitaxis (Armstrong *et al.* 2006), regulation of fat storage (Reis *et al.* 2010), starvation resistance (Harbison *et al.* 2004), cell size determination (Bjorklund *et al.* 2006), and mRNA alternative splicing (Brooks *et al.* 2015). SHEP proteins bind the *gypsy* insulator proteins SU(HW) and MOD(MDG4) and suppress chromatin insulator activity specifically in the nervous system (Matzat *et al.* 2012). The vertebrate orthologs of *shep*, which belong to the MSSP (*c-myc* single-strand-binding protein) family, encode proteins that complex with Myc/Max to inhibit E-box-based transcriptional activity (Niki *et al.* 2000b; Chen *et al.* 2014). MSSPs also regulate cell transformation, apoptosis, and DNA replication through interaction with Myc (Kimura *et al.* 1998; Niki *et al.* 2000a,b; Nomura *et al.* 2005), and they positively regulate TGFβ signaling during neural crest development (Jayasena and Bronner 2012).

Here, we take a modifier screening approach to identify mechanisms by which *shep* functions to regulate neuronal remodeling. In the absence of *a priori* models regarding a gene’s function, this approach can reveal strong molecular interactions that are critical to a given process (Ward *et al.* 2003; Kaplow *et al.* 2007; Kucherenko *et al.* 2008). Under the conditions used for this modifier screen, bursicon neuron-targeted *shep* RNA interference (RNAi) led to intermediate wing expansion defects and neuronal remodeling phenotypes that could be either enhanced or suppressed by introduction of genetic *shep* modifiers (Luan *et al.* 2006; Peabody *et al.* 2008; Zhao *et al.* 2008). By crossing 702 deficiency strains to a *shep* RNAi strain, we screened ∼86% of the *D. melanogaster* euchromatic genes and identified 24 regions containing candidate suppressors. Further cellular analysis narrowed the set to 12 deficiencies that suppressed defects in neurite morphology or soma growth of the bursicon neurons. By mapping with RNAi to individual loci, we successfully identified four suppressor genes [*CG10565*, *Myc*, *Olig family* (*Oli*), and *Daughters against dpp* (*Dad*)] that rescue the *shep*-dependent cellular defects of bursicon neurons. *Oli* and *Dad* were further confirmed as suppressors through crosses with independent mutant alleles. *Dad* encodes an inhibitory Smad protein (Kamiya *et al.* 2008), thus implicating an interaction between bone morphogenetic protein (BMP) signaling and *shep* in the remodeling process. Manipulation of the BMP receptor *tkv* suggested that BMP signaling is regulated by *shep* antagonism against *Dad* to control neuronal remodeling. Taken together, these findings shed light on the molecular mechanisms by which SHEP regulates postembryonic, structural plasticity of neurons.

## Materials and Methods

### Stocks

*D. melanogaster* stocks and crosses were cultured on standard cornmeal-yeast-agarose media at 25° unless otherwise noted. We obtained 702 Exelixis, DrosDel, and Bloomington Stock Center Deficiency Project (BSC) deficiency strains for the X, second, and third chromosomes from the Bloomington *Drosophila* Stock Center. Based on the deficiency breakpoints and gene locations (Cook *et al.* 2012), we calculated that these deficiencies covered 86% of the euchromatic genes in the genome.

Most RNAi strains were obtained from the KK collection at the Vienna *Drosophila* RNAi Center. The *su(Hw)* RNAi strain (P{GD4493}v10724; FBti0091830), *UAS-su(Hw)*, and alleles for the *su(Hw)* gene came with a genetic *y^2^ct^6^* background as generous gifts from Elissa Lei [National Institute of Diabetes and Digestive and Kidney Diseases (NIDDK), Bethesda, MD] (Matzat *et al.* 2012). The other strains used included *386-Gal4* (w*; *P{GawB}386Y*; FBti0020938) (Bantignies *et al.* 2000), *tub-Gal80^ts^* (w*; *P{tubP-GAL80ts}2*; FBst0007017) (Ferris *et al.* 2006), *burs-Gal4* (*w*; *bursicon-Gal4*) (Peabody *et al.* 2008), *UAS-Dcr-2* (*w[1118]*; *P*{*UAS-Dcr-2*, *w*[+]}; FBst0024650), *UAS-shep-RNAi* (*w[1118]*; *P{GD5125}v37863*; FBti0092714), *ccap-Gal4* (y* w*; *P*{*ccap-Gal4.P*}*16*; FBti0037998) (Park *et al.* 2003), *UAS-tkv^Q199D^*, and *UAS-tkv^Q253D^* (gifts from Michael B. O’Connor, University of Minnesota).

### Screen crosses and scoring

All deficiencies on the second chromosome were balanced with *CyO*, *Act-GFP* (FBst0004533). Deficiencies on the third chromosome were balanced with *TM6B*, *Tb^1^*. For deficiencies on the second and third chromosomes and RNAi strains, five males were crossed with 16 w*/w^1118^, *UAS-shep-RNAi*, *UAS-Dcr-2*; *386-Gal4*, *tub-Gal80ts* (386 > *shep-RNAi*, *Dcr-2*, *tub-Gal80^ts^*) virgin females and kept at 30° on regular food. On day 4 after the cross, the parents were removed, and the progeny were scored on days 10, 12, and 14. For deficiencies on the X chromosome, 20 virgin females from each deficiency stock were crossed to five 386 > *shep-RNAi*, *Dcr-2*, *tub-Gal80^ts^* males. Along with every round of crosses, we included a control cross of 386 > *shep-RNAi*, *Dcr-2*, *tub-Gal80^ts^* to the isogenic *w^1118^* background stock that was used to create the DrosDel deficiencies. Wing expansion was scored for at least 20 flies. Test crosses were repeated for another two rounds for the strongest 50 suppressor deficiencies.

### Immunostaining and imaging

Immunostaining was performed as previously described (Hewes *et al.* 2003). We used antibodies against the α-subunit of the bursicon protein (anti-BURS) (1:5000, PFA/PA) (Luan *et al.* 2006) to determine cellular phenotypes of bursicon neurons. Secondary antibodies conjugated with Cy3 or ALEXA 488 from goat and mouse were each used at a 1:500 dilution. Cells and projections were imaged as confocal z-series scans with an Olympus (Center Valley, PA) FluoView FV500 confocal microscope, and a Leica (Mannheim, Germany) SP8 scanning multiphoton microscope. Identical settings were used in parallel for all samples in each experiment. For the B_AG_ neurons at the P14 pharate adult stage, we measured the average soma area of the six most anterior neurons in the abdominal ganglia of each preparation as previously described (Chen *et al.* 2014). Axonal branches of the B_AG_ neurons were counted in Adobe Illustrator by Sholl analysis (Milosevic and Ristanovic 2007) with concentric circles spaced 50 μm apart. The area covered by the ventral portion of the B_SEG_ arbor within the subesophageal ganglia (Figure 2J, magenta; Supplemental Material, File S1 and File S3) was imaged as maximum intensity z-series projections, and was measured by first setting an image threshold pixel intensity of 40 in Fiji (Schindelin *et al.* 2012) and then counting all above-threshold pixels. Three dimensional tracings were made of z-series scans with the filament function in Imaris (South Windsor, CT).

### Data availability

The authors state that all data necessary for confirming the conclusions presented in the article are represented fully within the tables and figures. All fly strains are available upon request.

## Results

### A sensitized loss-of-*shep* background for genetic modifier screening

**Figure 1:**
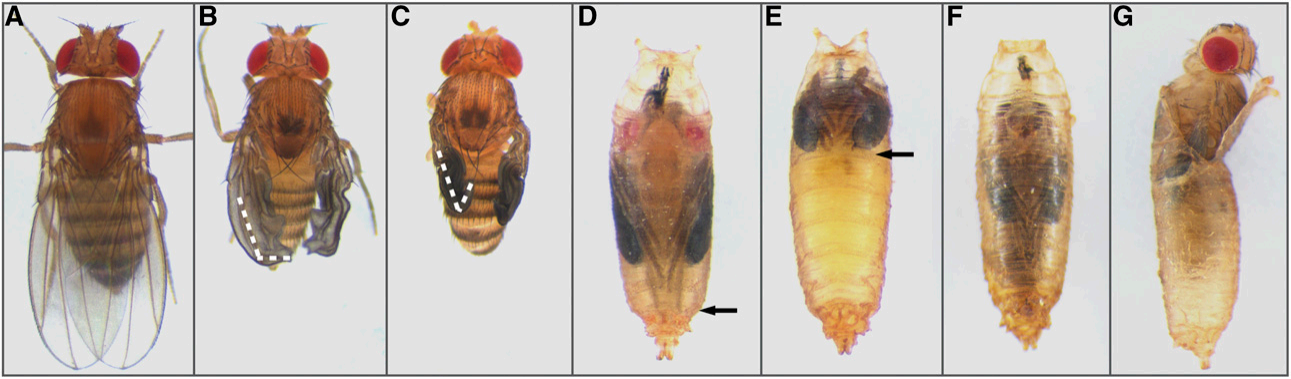
Wing expansion defects and lethality produced by loss of *shep* in peptidergic neurons. (A–C) Three wing expansion categories for modifier screen scoring. EXW (A), fully expanded wings. UEW (C), wings with the distal tip opened < 90° relative to the long axis of the wing (white dashed line) were scored as unexpanded. PEW (B), all wings that opened > 90° (white dashed line) but were not flattened were scored as partially expanded. (D) Pupa with normal head eversion. The arrows in (D) and (E) indicate the distal tips of the metathoracic legs. (E) Pupa with head eversion defects: the head in this pupa remained entirely within the thorax, and the wings and legs were not extended toward the posterior. (F) Pupa that displayed late pupal lethality, marked by pigmentation of the eyes and wings (later than stage 12) (Bainbridge and Bownes 1981) and subsequent desiccation of the animal after death. (G) Some flies initiated eclosion, often freeing their prothoracic legs, but then died after failing to completely exit the pupal case.

The *shep* gene regulates neuronal remodeling during metamorphosis. Loss of *shep* leads to defects in a neuropeptide-regulated behavior, wing expansion, which is easy to score and thus suitable as a readout for genetic screens (Chen *et al.* 2014). To create a sensitized loss-of-*shep* background for genetic modifier screening, we tested multiple Gal4 lines for driving expression of *UAS-shep-RNAi* and *UAS-Dcr-2*. These included the pan-neuronal driver *elav-Gal4*, the bursicon neuron-specific driver *burs-Gal4*, and the peptidergic neuron driver *386-Gal4* (Bantignies *et al.* 2000; Taghert *et al.* 2001). The *shep* RNAi driven by *elav-Gal4* and *burs-Gal4* led to severe pupal lethality and weak wing expansion defects, respectively. The *386-Gal4*-driven *shep* RNAi generated viable flies with the moderate wing expansion defects (see below) needed in a loss-of-*shep* background suitable for detecting genetic modifiers. We included a temperature-sensitive *Gal4* inhibitor, *tub-Gal80^ts^*, in the test stock, 386 > *shep-RNAi*, *Dcr-2*, *tub-Gal80^ts^*, to minimize the phenotypic drift that we have sometimes observed in permanent wing expansion defective stocks. This test stock grew and bred normally, with normal wing expansion in homozygotes at 25°. When the test stock flies were crossed at 30° to the isogenic *w^1118^* background stock (hereinafter referred to as “control A” stock) that was used to create the DrosDel deficiencies, the heterozygous progeny displayed 16% fully expanded wings, 40% partially expanded wings, and 44% unexpanded wings (Figure 1, A–C). Crosses to the test stock provided a sensitized background to select for suppressors or enhancers of the wing expansion phenotype.

To verify the sensitivity of this screen system to *shep* interactions, we knocked down the *gypsy* chromatin insulator factor *su(Hw)* and measured the effects on wing expansion. The *gypsy* insulator proteins are the only known *shep*-interacting factors, and *shep* inhibits *gypsy* insulator activities specifically in the nervous system (Matzat *et al.* 2012). After crossing the test stock 386 > *shep-RNAi*, *Dcr-2*, *tub-Gal80^ts^* with *UAS-su(Hw)-RNAi* at 30°, we detected suppression of the *shep* RNAi wing expansion defects (Figure S1A). To control for potential off-target effects of the *su(Hw)* RNAi, two deletion alleles, *su(Hw)^V^* (FBal0032826) (Harrison *et al.* 1992) and *su(Hw)^tHa^* (FBal0046546) (Harrison *et al.* 1992), and one insertion allele *su(Hw)^2^* (FBal0016319) (Parkhurst *et al.* 1988), were crossed to the test stock at 30°. We detected suppression of wing expansion defects by either *su(Hw)^tHa^* or *su*(*Hw*)*^V^* alone (Figure S1A), and *su(Hw)^2^* displayed a trend toward suppression of the wing expansion defects (Fisher’s exact test, *P* = 0.056). Conversely, we observed enhancement of the *shep* loss-of-function phenotype when we crossed the test stock flies with *UAS-su(Hw)* flies at 30°. The overexpression of *su(Hw)* together with *shep* RNAi led to 30% pupal lethality (*n* = 104), with 45% of these pupae also displaying defective head eversion, which is a phenotype associated with earlier disruptions of bursicon neurons and other peptidergic cells in the *386-Gal4* pattern (Zhao *et al.* 2008). Crosses with the genetic background strain produced only 4% lethality (*n* = 75). These tests verified that the *shep* RNAi test stock provided a sensitized background for detecting suppressors and enhancers of a *shep* loss-of-function phenotype that could be readily scored in a high-throughput genetic screen.

**Figure 2:**
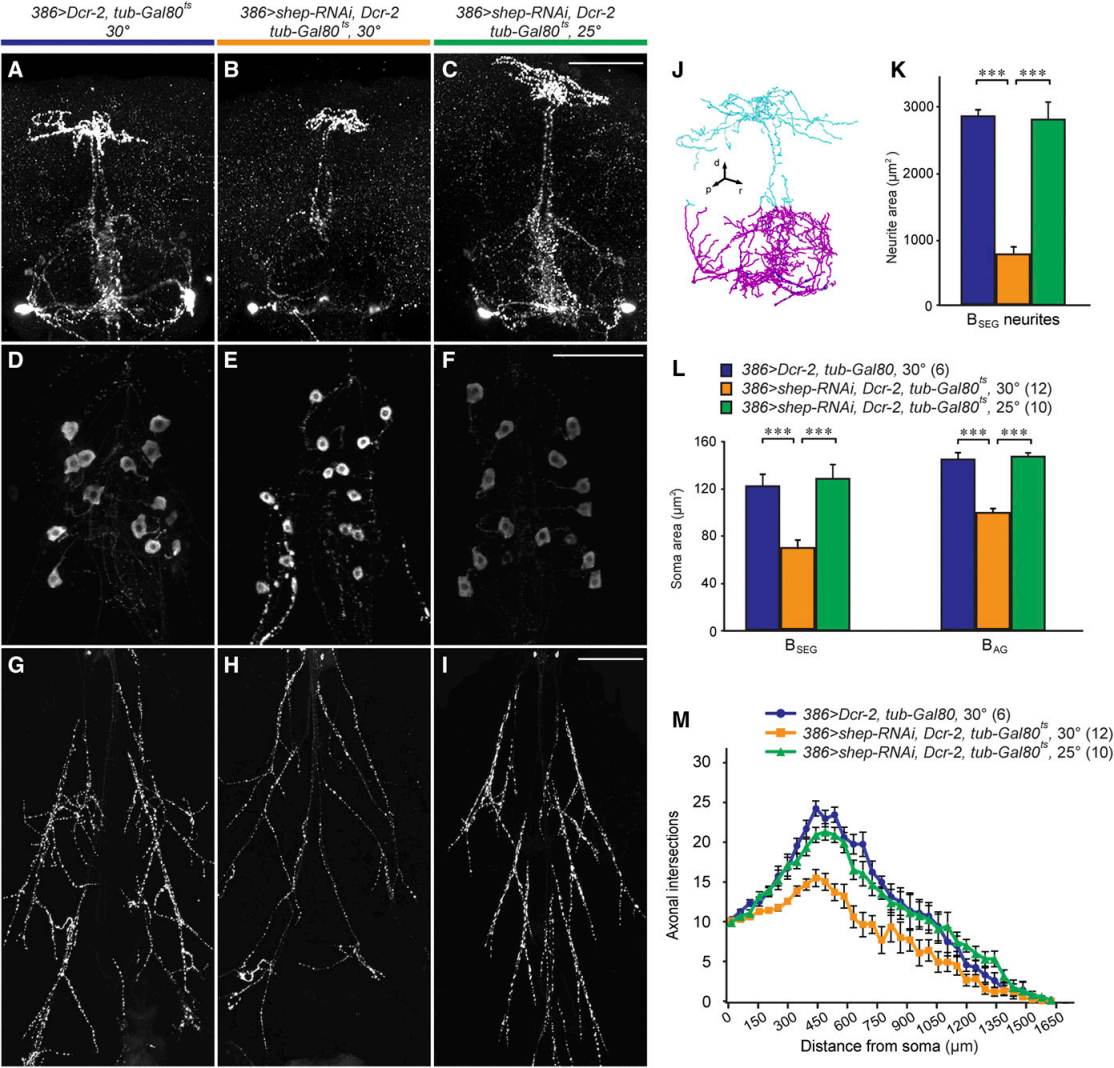
Loss of *shep* reduced soma area and neurite branching at the P14 pharate adult stage. (A–C) Anti-BURS (bursicon protein) immunostaining showed the effects of *shep* RNA interference (RNAi) on neurites of the B_SEG_ neurons in the subesophageal ganglia. Pharate adult 386 > *shep-RNAi*, *Dcr-2*, *tub-Gal80^ts^* animals displayed reduced neurite branching at 30° (B), the restrictive temperature for Gal80^ts^, but not at 25° (C), the permissive temperature. (A) A genotype control without *shep* RNAi at the restrictive temperature. Bar, 100 µm. (D–F) Reduced soma areas were observed in the B_AG_ neurons of the 386 > *shep-RNAi*, *Dcr-2*, *tub-Gal80^ts^* animals at 30°, but not at 25° (F) or in non-RNAi controls at 30° (D). Bar, 100 µm. (G–I) We observed reduced branching in the B_AG_ neuron peripheral axon arbor of a 386 > *shep-RNAi*, *Dcr-2*, *tub-Gal80^ts^* pharate adult at 30° (H), but not in the temperature (I) or genotype (G) controls. Bar, 200 µm. (J) A 3D tracing (projected to 2D) showing the organization of the B_SEG_ neurite arbor in the brain (cyan) and subesophageal ganglia (magenta). Maximum intensity projection images of the ventral portion of the B_SEG_ neurite arbor (magenta) were used for B_SEG_ neurite area quantification. d, dorsal; p, posterior; r, right. (K) Quantification of the area covered by the magenta portion of the B_SEG_ arbor for the genotypes shown in (A–C). *P* < 0.0000001, one-way ANOVA [***P < 0.001, Tukey’s HSD (honest significant difference) *post hoc* test]. Sample sizes were the same as in (L) and (M). (L) Quantification of B_SEG_ and B_AG_ neuron soma areas for P14 pharate adults. One-way ANOVAs were done for the B_SEG_ and B_AG_ somata separately (*P* < 0.000001; Tukey’s HSD *post hoc*, ***P < 0.001; sample sizes in parentheses). (M) Sholl analysis of branches in the B_AG_ peripheral axon arbors. The space between each of the concentric rings used to count intersecting axons was 50 μm.

The bursicon neurons, which are a subgroup of neurons covered by the *386-Gal4* driver (Figure S1, B–D), undergo extensive remodeling during metamorphosis that consists of relocation and enlargement of cell bodies as well as pruning and regrowth of neurites (Zhao *et al.* 2008). We have previously shown that loss of *shep* leads to reduced soma and neurite growth and reduced neurite branching in these neurons (Chen *et al.* 2014). The bursicon neurons located in the subesophageal ganglia (B_SEG_ cells) play an essential command role upstream of bursicon neurons in the abdominal ganglia (B_AG_ cells), which secrete bursicon into the blood to control associated posteclosion events (Luan *et al.* 2006; Peabody *et al.* 2008). Thus, disruption of the remodeling of the bursicon neurons during metamorphosis often results in disturbance of adult cuticle tanning and wing expansion (Luan *et al.* 2006; Zhao *et al.* 2008; Gu *et al.* 2014). At the P14 pharate adult stage, B_SEG_ and B_AG_ cells in progeny from crosses of the control A stock to 386 > *shep-RNAi*, *Dcr-2*, *tub-Gal80^ts^* at 30° displayed smaller soma areas revealed by anti-BURS immunostaining (Figure 2, A–F and L). In addition, the B_SEG_ cells had a less profuse CNS arbor (Figure 2, A–C and K), and the B_AG_ cells had fewer neurite projections in the peripheral axon arbor (Figure 2, G–I and M). By contrast, bursicon cell morphologies were normal in the same crosses at 25° (Figure 2). Since bursicon neurons undergo dramatic enlargement of soma sizes and extension of new projections during the neuronal outgrowth phase (Zhao *et al.* 2008), these morphological defects likely result from defective outgrowth upon loss of *shep*, but detailed time-series analysis will be required to assess whether *shep* also promotes the maintenance of neurites after the completion of outgrowth. Taken together, these tests verified that the sensitized background used here for detecting *shep* modifiers of wing expansion displayed the neuronal remodeling defects seen previously with other *shep* loss-of-function genotypes.

### Deficiency screen for *shep* modifiers

A modifier screen was conducted with 702 deficiency strains from the DrosDel (Ryder *et al.* 2004), Exelixis (Parks *et al.* 2004), and BSC (Cook *et al.* 2012) collections that covered 86% of the genes in the euchromatic genome. These deficiencies were crossed to the test stock at 30°, and deficiencies that deleted *shep*-interacting factors were expected to modify (by either enhancing or suppressing) the wing expansion defects. A total of 69 crosses resulted in pupal lethality (Figure 1, E–G). These deficiencies were classified as enhancers (Table S1 in File S2), and were not further investigated.

**Figure 3:**
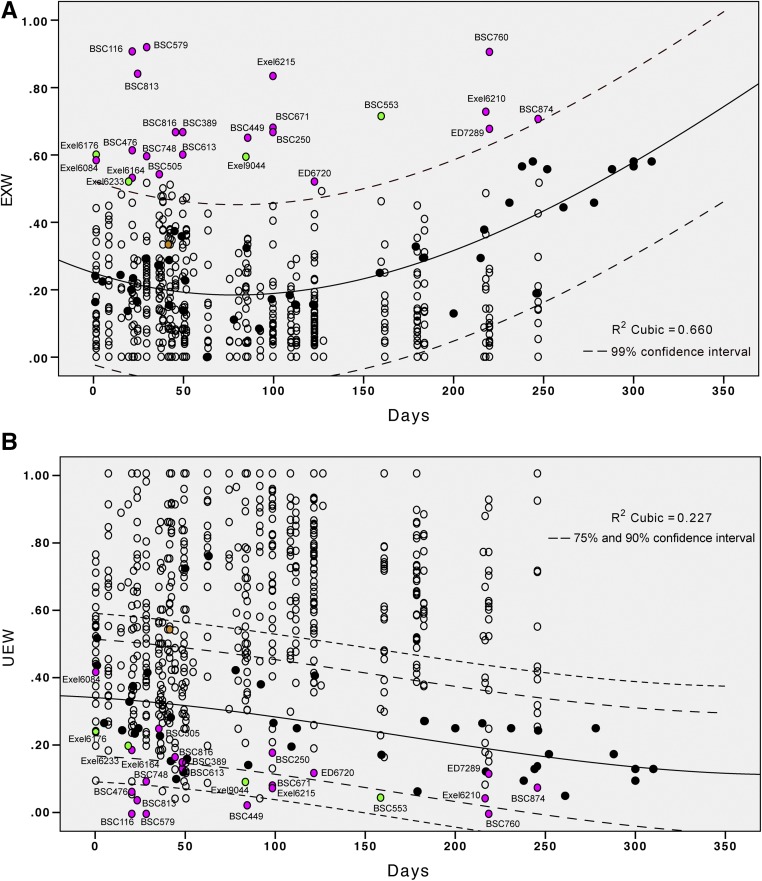
Plots of wing expansion scores for all deficiencies that produced adult progeny. (A) Percentage of EXW (fully expanded wings) progeny for all deficiencies. Black-filled circles, EXW scores for control crosses (the test stock crossed to the control A stock) were plotted as a function of time (in days) after the test stock was created. Open circles, percentage of EXW progeny in the test crosses (386 > *shep-RNAi*, *Dcr-2*, *tub-Gal80^ts^* test stock crossed to individual deficiencies). Magenta-filled, EXW percentages for suppressor deficiencies that were selected for further analysis but not mapped to individual suppressor genes. Green-filled circles, EXW percentages for suppressor deficiencies that were successfully mapped to individual genes for tests of cellular rescue. Orange-filled circle, EXW percentage for a deficiency that deleted the *su(Hw)* gene. The line of best fit with 99% C.I. was generated with the cubic method in SPSS. (B) A similar plot of UEW (wings with the distal tip opened < 90° relative to the long axis of the wing) scores for all deficiencies.

For all other crosses in which adult progeny emerged, we scored the degree of wing expansion as expanded wings (EXW), partially expanded wings (PEW), and unexpanded wings (UEW) (Figure 1), and scores were summarized for 633 deficiencies that produced adult progeny (Figure S2). In spite of the *tub-Gal80^ts^* inhibitor in the test stock, we detected phenotypic drift (manifest as higher percentages of PEW and EXW progeny in the control crosses) in two test stocks that we generated sequentially and used eight months apart (Figure S3). To control for phenotypic drift, we obtained the lines of best fit plotted for EXW and separately for UEW as a function of time (in days). Among 24 suppressor deficiencies with the highest EXW scores (above the 99% C.I.), we observed inversely related EXW and UEW scores (represented by magenta and green dots in Figure 3). Four of these deficiencies were eliminated for further tests when other deficiencies failed to reproduce the suppression, or due to potential effects on the efficacy of RNAi (Table S2 in File S2). Three deficiencies deleted the same regions as other members of the set of 24 candidate suppressors (Table S2 in File S2). Therefore, we narrowed down the list of candidate suppressors to 17 regions, each containing 1–20 genes (Table S2 in File S2).

**Figure 4:**
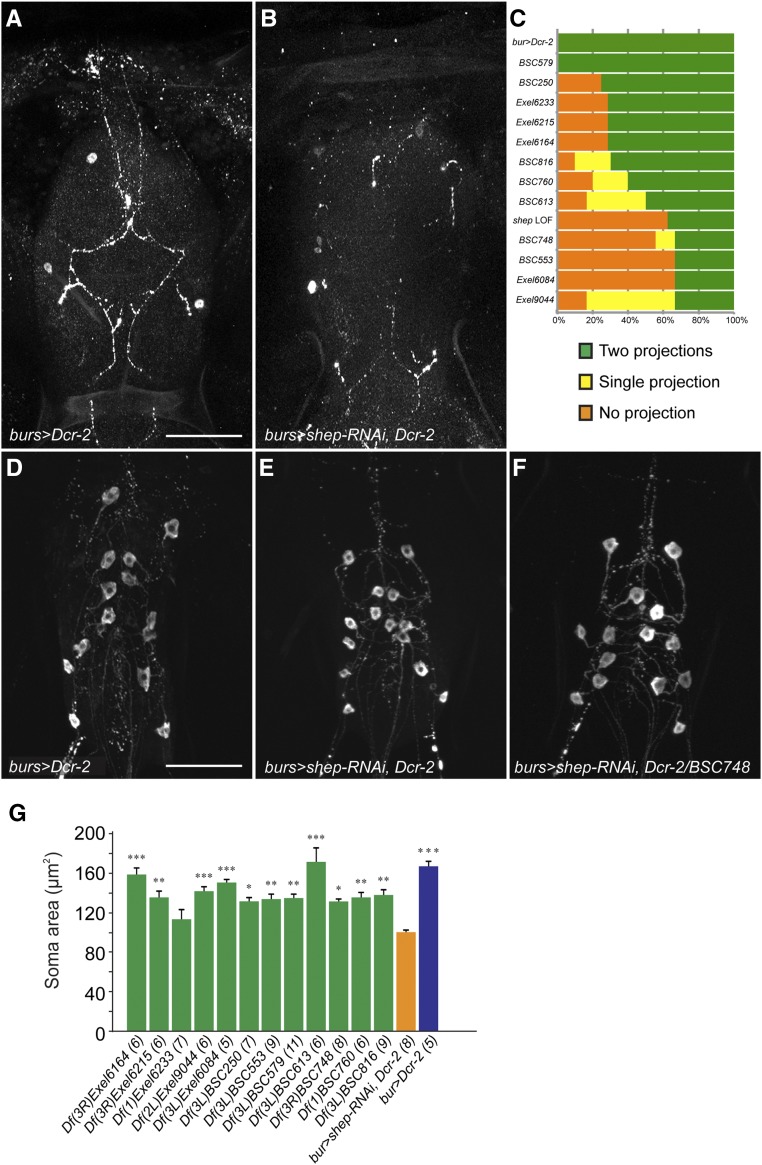
Suppression of bursicon neuron phenotypes by selected deficiencies. (A and B) Anti-BURS (bursicon protein) immunostaining detected projections in the thoracic ganglia (A) that originated from B_SEG_ neurons in *burs* > *Dcr-2* animals. These projections were mostly absent in *burs* > *shep-RNAi*, *Dcr-2* animals (B). Bar, 100 µm. (C) Counts of B_SEG_ neuron projections in the thoracic ganglia of progeny from crosses with *burs* > *shep-RNAi*, *Dcr-2* to suppressor deficiencies. Phenotypes of the B_SEG_ projections were divided into three categories (orange, yellow, or green), depending on whether none, one, or both B_SEG_ neurites were detected by anti-BURS immunostaining in the thoracic ganglia. *shep* LOF, *shep* loss-of-function progeny from the cross of *burs* > *shep-RNAi*, *Dcr-2* to control A. (D–F) Rescue of B_AG_ soma areas by suppressor deficiencies. The soma area was reduced in *burs* > *shep-RNAi*, *Dcr-2* animals at the P14 pharate adult stage (E), and this defect was rescued by crossing *burs* > *shep-RNAi*, *Dcr-2* to suppressor deficiencies [as shown for *Df(3R)BSC748* in (F)]. Bar, 100 µm. (G) Quantification of the mean B_AG_ soma areas in test crosses with suppressor deficiencies. The B_AG_ neuron soma areas were smaller in *burs* > *shep-RNAi*, *Dcr-2* P14 pharate adults (orange column) than in the control, *burs* > *Dcr-2* animals (blue column). A total of 11 of the 17 suppressor deficiencies produced significant rescue of the soma areas (green columns). *P* < 0.000001, one-way ANOVA (* *P* < 0.05, ** *P* < 0.01, *** *P* < 0.001, Tukey’s *post hoc* test; sample sizes in parentheses).

We have previously identified several genes that produce wing expansion defects when misexpressed in the *386-Gal4* pattern but not the more restricted *ccap-Gal4* pattern, which contains the bursicon neurons (Zhao *et al.* 2008). Therefore, some suppressors may reflect interactions within other nonbursicon neurons that regulate wing expansion. To test whether the deficiency suppressors of the wing expansion phenotype also suppressed the bursicon neuron cellular phenotype, we performed anti-BURS immunostaining on *burs* > *UAS-shep-RNAi*, *Dcr-2* animals that were crossed to the 17 suppressor deficiencies. None of the 17 deficiencies rescued the number of peripheral bursicon axonal branches (data not shown). Nevertheless, with 12 of the 17 deficiencies, we observed rescue of the B_SEG_ cell neurite projections to the thoracic ganglia (Figure 4, A–C) and/or restoration of B_AG_ neuron soma size (Figure 4, D–G and Table S2 in File S2). Therefore, these 12 deficiencies were retained for further investigation.

We expected to find some deficiencies that nonspecifically produced suppression by reducing the efficacy of transgene expression in the bursicon neurons (*e.g.*, by interfering with Gal4 expression). In addition, we were less interested in deficiencies that promoted bursicon neuron growth independently of *shep* (*i.e.*, in a *shep* wild-type background). To rule out deficiencies with such nonspecific genetic interactions, we monitored soma areas and the expression levels of a membrane-tagged GFP reporter (Figure S4C arrows) in *ccap* > *mCD8*::*GFP/deficiency* animals. None of the 12 suppressor deficiencies led to changes in GFP levels (Figure S4D), which would have reflected changes in efficacy of the Gal4-UAS expression system. In addition, none of these deficiencies promoted bursicon neuron cellular growth by themselves (Figure S4E), indicating that the suppressor deficiencies rescued the wing expansion performance and cellular phenotypes by interacting specifically with *shep*. There were three deficiencies that significantly reduced soma size, and the fact that they still acted as suppressors suggests stronger suppression of *shep* function by these deficiencies than the others.

### RNAi-based modifier screen for *shep* suppressor genes

**Figure 5:**
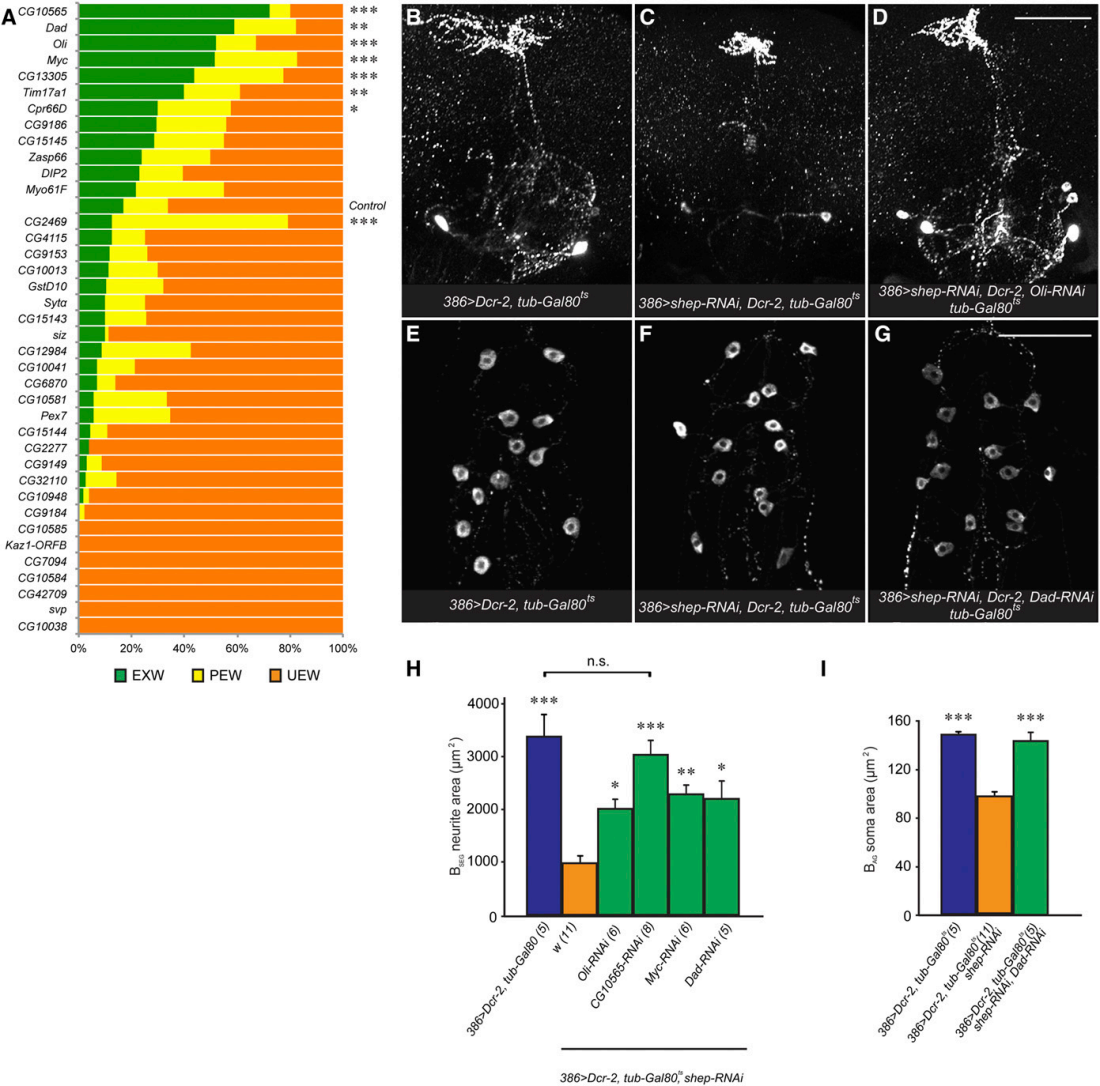
Mapping of candidate *shep* suppressor genes by RNA interference (RNAi). (A) Wing expansion rates obtained in crosses of the *shep* RNAi test stock (386 > *shep-RNAi*, *Dcr-2*, *tub-Gal80^ts^*) to RNAi for genes contained within nine selected suppressor deficiencies. The histogram shows a plot of wing expansion scores for each of these crosses. * *P* < 0.05, ** *P* < 0.01, and *** *P* < 0.001 (*n* ≥ 20, Fisher’s exact test with Bonferroni correction, with test crosses compared to the control). (B–D) RNAi-mediated rescue of the B_SEG_ neurites. Loss of *shep* led to a reduced B_SEG_ neurite arbor in the subesophageal ganglia (C). Loss of suppressor genes partially or completely restored this arbor; (D) shows rescue of the B_SEG_ neurons by *Oli* RNAi. Bar, 100 µm. (E–G) RNAi of suppressor genes rescued B_AG_ neuron soma areas. Loss of *shep* in 386 > *shep-RNAi*, *Dcr-2*, *tub-Gal80^ts^* animals led to smaller B_AG_ neuron soma areas [(F); quantification in (I)] than in control animals (E). This defect was rescued by *Dad* RNAi (G). Bar, 100 µm. (H) Loss of suppressor genes rescued B_SEG_ cell neurite areas at 30°. The 386 > *shep-RNAi*, *Dcr-2*, *tub-Gal80^ts^* animals showed significantly fewer B_SEG_ neurites (orange) than the 386 > *Dcr-2*, *tub-Gal80^ts^* control animals (blue). Introduction of RNAi for four suppressor genes into the 386 > *shep-RNAi*, *Dcr-2*, *tub-Gal80^ts^* animals produced partial to complete (*CG10565* RNAi) rescue (green). The data (sample sizes in parentheses) were analyzed with a one-way ANOVA (*P* = 0.000001) with Tukey’s HSD (honest significant difference) *post hoc* tests compared to the control genotype (orange) or between genotypes indicated with a bracket; * *P* < 0.05, ** *P* < 0.01, and *** *P* < 0.001; n.s., not significant. (I) Quantification of B_AG_ neuron soma areas with or without *Dad-RNAi*. *P* < 0.000001, one-way ANOVA (*** *P* < 0.001, Tukey’s HSD *post hoc*).

To map the genetic interactions uncovered by the above deficiencies to single loci, we selected 9 of the 12 suppressor deficiencies (Table S2 in File S2) for RNAi-based modifier screening, based on the availability of reagents for genes directly adjacent to or deleted by these deficiencies. Of 45 tested RNAi strains, eight provided statistically significant suppression of wing expansion defects in crosses to the 386 > *shep-RNAi*, *Dcr-2*, *tub-Gal80^ts^* strain at 30° (Figure 5A). We focused on the strongest four suppressors, *CG10565*, *Dad*, *Oli*, and *Myc*, as the best candidates for cellular-level analysis. RNAi strains that displayed no suppression (Figure 5A) or lethality (Table S3 in File S2) were not further investigated.

To test whether these four suppressor RNAi strains rescued the wing expansion defects by affecting the development of the bursicon neurons, we examined the morphology of the B_AG_ and B_SEG_ neurons of the progeny from the same crosses referred to above. Consistent with the results of deficiency-based suppression (Figure 4, A–C), the RNAi-based suppression was largely detected in the B_SEG_. The four RNAi strains targeting *Dad*, *Myc*, *Oli*, or *CG10565* all restored B_SEG_ neurite projections in the subesophageal ganglia (Figure 5, B–D and H). However, none of the four RNAi lines rescued branching in the peripheral axon arbor (data not shown), and only the RNAi to *Dad* restored B_AG_ neuron soma areas (Figure 5, E–G, and I). Thus, loss of *CG10565*, *Dad*, *Oli*, and *Myc* suppressed the effects of *shep* knockdowns on wing expansion, and in each case the effects were associated with rescue of adult B_SEG_ neurites in the subesophageal ganglia.

**Figure 6:**
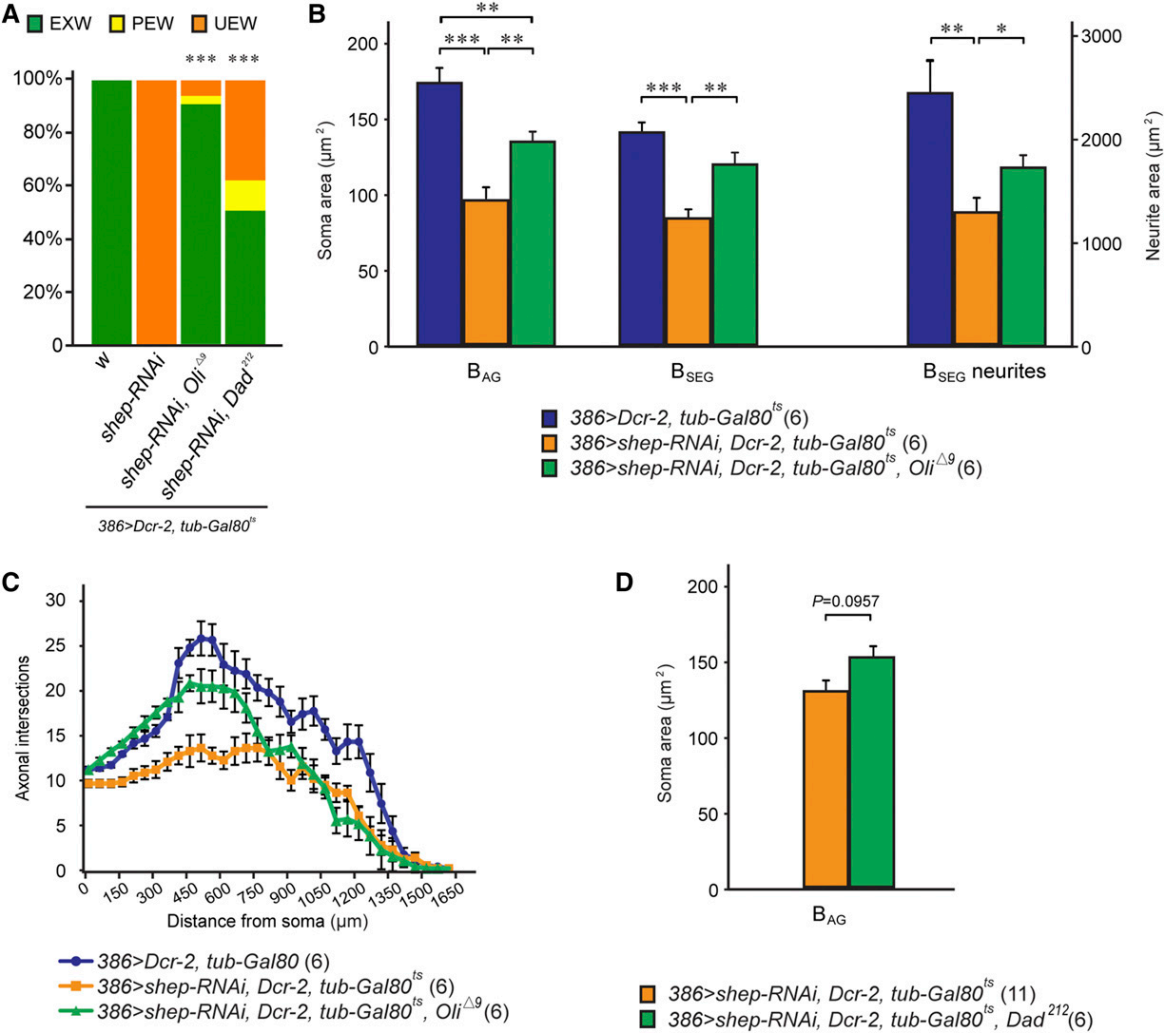
Suppression of wing expansion defects and bursicon cellular defects by *Dad* and *Oli* alleles. (A) Heterozygous *Oli* and *Dad* alleles suppressed the wing expansion defects caused by *shep* RNA interference (RNAi). Wing expansion scores for the genotypes shown were analyzed with the Fisher’s exact test with Bonferroni correction, *** *P* < 0.001. EXW, expanded wings; PEW, partially expanded wings; UEW, unexpanded wings. (B) The *Oli*^Δ^*^9^* mutant allele partially restored the cellular defects seen in bursicon neurons with *shep* RNAi. One-way ANOVA (*P* = 0.000017, 0.00005, and 0.00217 for B_AG_ and B_SEG_ soma sizes and B_SEG_ neurites, respectively) with Tukey’s honest significant difference (HSD) *post hoc* tests, * *P* < 0.05, **P < 0.01, and *** *P* < 0.001. (C) Sholl analysis of B_AG_ peripheral axon arbors. When crossed to the 386 > *shep-RNAi*, *Dcr-2*, *tub-Gal80^ts^* test stock, the *Oli*^Δ^*^9^* mutant allele partially rescued B_AG_ peripheral axon branching defects caused by loss of *shep*. (D) B_AG_ cell soma size following attempted rescue with a heterozygous *Dad* allele (Student’s *t*-test, *P* = 0.0957).

We performed four additional control experiments to validate the candidate suppressors. First, we tested whether the suppression caused by RNAi for *Dad*, *Oli*, and *Myc* could be phenocopied with independent loss-of-function alleles for each gene (no independent alleles were available for *CG10565*). We crossed the test stock to *Dad^212^* (Ogiso *et al.* 2011), *Myc^2^* (Maines *et al.* 2004), *Myc^4^* (Pierce *et al.* 2004), *Oli*^Δ^*^9^* (Oyallon *et al.* 2012), and their respective genetic background strains at 30°. *Dad^212^*, *Myc^4^*, and *Oli*^Δ^*^9^* contain small deletions and are likely molecular null alleles, and *Myc^2^* is the result of a point mutation and is a strong, homozygous lethal allele. *Dad^212^* or *Oli*^Δ^*^9^* produced strong suppression of the wing expansion defects, with 50% EXW (*n* = 26) and 91% EXW (*n* = 32) among the heterozygous progeny, respectively (Figure 6A). In contrast, control crosses with *yw* or *y^1^w^67c23^* (Toba *et al.* 1999), the genetic background strains for *Dad^212^* (Ogiso *et al.* 2011) and *Oli*^Δ^*^9^*, respectively, both produced progeny with 100% UEW rates. The *Oli*^Δ^*^9^* allele also partially rescued soma areas and neurite arbors in both the B_AG_ and B_SEG_ neurons (Figure 6, B and C). These results confirmed that the suppression of *shep* phenotypes was due to loss of *Oli* rather than off-target effects of the RNAi. The *Dad^212^* allele rescued the wing expansion defects, and there was also a trend (*P* = 0.0957) (Figure 6D) suggesting that *Dad^212^* had a positive effect on the soma areas of B_AG_ neurons. This may simply reflect stronger knockdown of *Dad* expression by the *Dad* RNAi, which led to suppression of both the wing expansion defects and cellular defects of bursicon neurons, than by the heterozygous *Dad^212^* allele. In contrast, crosses of the test stock with *Myc^2^* and *Myc^4^* failed to suppress the wing expansion phenotype, and *Myc^4^* failed to suppress the cellular phenotypes observed in the bursicon neurons (data not shown). Therefore, we eliminated *Myc* from further consideration, although we cannot rule out *Myc* as a suppressor of *shep*, since RNAi may produce a stronger knockdown than a heterozygous null allele.

In a second set of control experiments, we tested whether the candidate suppressors altered the morphology of bursicon neurons in the absence of *shep* RNAi. Crosses of 386 > *Dcr-2*, *tub-Gal80^ts^* animals to the *Dad* RNAi strain or the *Oli*^Δ^*^9^* allele at 30°, respectively, both produced progeny with normal wing expansion and cellular morphology (Figure S5, A–C). Therefore, the observed suppression of cellular defects in bursicon neurons was not caused by loss of *Dad* or *Oli*, but relied on genetic interactions between *shep* and these two genes. Crosses of 386 > *Dcr-2*, *tub-Gal80^ts^* animals to the *CG10565* RNAi strain produced smaller B_AG_ soma size, and fewer B_AG_ neuron peripheral branches (Figure S5, C and D). However, *CG10565* RNAi did not alter the morphology of the B_SEG_ neurites (Figure S5C), where the genetic interaction between *CG10565* RNAi and *shep* RNAi was detected (Figure 5H). The latter result implicates *CG10565* as a true suppressor of *shep*, although that conclusion is tempered by the other effects of *CG10565* RNAi alone on the B_AG_ and B_SEG_ neurons. Therefore, we did not investigate *CG10565* further.

In the third control, we tested whether *Oli* and *Dad* suppressed the *shep* RNAi phenotype simply by reducing Gal4-UAS transgene expression. We measured this indirectly by assessing the impacts of these genotypes on levels of Gal4-dependent mCD8::GFP expression (fluorescence). With *UAS-mCD8*::*GFP*, *UAS-Dcr-2* expressed under the control of a *bursicon-Gal4* driver at 30°, the levels of mCD8::GFP fluorescence in the B_SEG_ and B_AG_ somata were the same with *shep* RNAi alone and with *shep* RNAi together with *Dad* RNAi or *Oli*^Δ^*^9^* (Figure S5E).

We have shown in previous research that peripheral neurites of the B_AG_ neurons could be resolved equally well with anti-BURS immunostaining as with the mCD8::GFP membrane tag (Chen *et al.* 2014). Similarly, we performed anti-BURS immunostaining in heterozygous *bursicon* > *shep-RNAi*, *Dcr-2*, *mCD8*::*GFP* animals, with or without *Oli*^Δ^*^9^*, that were raised at 30°. While the labeling was qualitatively different, with anti-BURS immunostaining favoring the labeling of boutons and mCD8::GFP labeling all plasma membrane, the resolution of gross neurite morphology was equivalent with these two markers (Figure S6). Therefore, the changes in the B_SEG_ arbor measured with anti-BURS immunostaining reflected changes in neurites. Taken together, our results confirmed *Dad* and *Oli* as suppressors of wing expansion defects and cellular phenotypes resulting from the loss of *shep* function.

### Suppression of loss-of-*shep* phenotypes relied on proper activation of the BMP signaling pathway

The identification of *Dad* as a *shep* suppressor implicated the BMP signaling pathway in *shep*-dependent neuronal remodeling during metamorphosis. The *Dad* gene encodes inhibitory Smad proteins (I-Smad) that physically interact with the BMP type I receptors, Sax and Tkv, and inhibit BMP signaling by interfering with Mad phosphorylation and dimerization with Medea (Inoue *et al.* 1998; Kamiya *et al.* 2008). Therefore, we hypothesized that *shep* antagonizes *Dad* to regulate BMP signaling, and loss of *shep* results in hyperactive *Dad* inhibition of the BMP signaling pathway. To monitor activation of the BMP signaling pathway in these neurons, we performed anti-pMad immunostaining. We detected heterogeneous pMad expression in the CNS, and the labeled cells included bursicon neurons. However, the levels of pMad varied dramatically during development and among similarly staged animals of the same genotype (data not shown). This variation precluded effective use of anti-pMad immunostaining to test our model.

**Figure 7:**
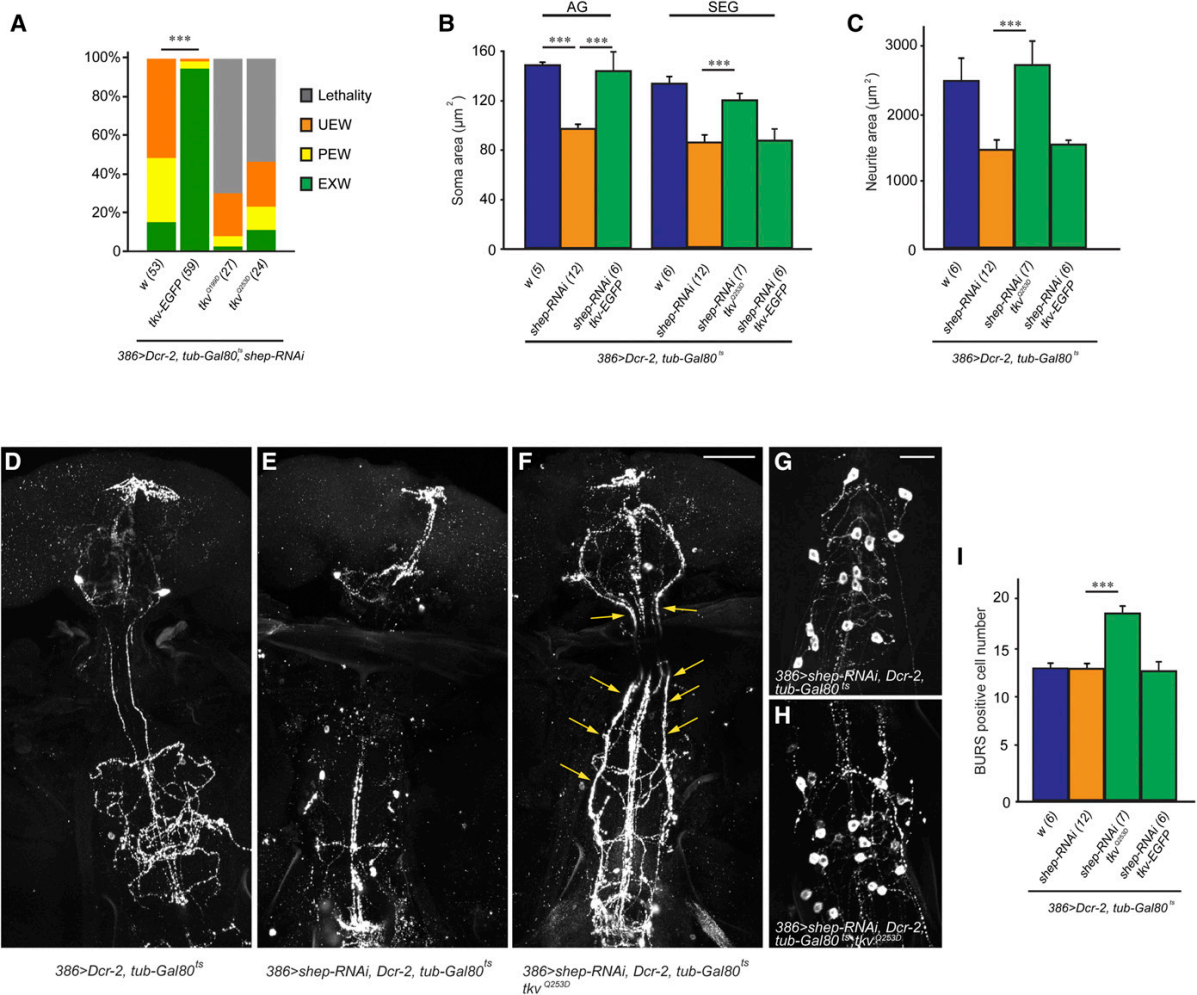
Activation of bone morphogenic protein (BMP) signaling pathway suppressed loss-of-*shep* phenotypes. (A) Expression of wild-type *tkv* (*UAS-tkv-EGFP*) in 386 > *shep-RNAi*, *Dcr-2*, *tub-Gal80^ts^* flies significantly rescued wing expansion. Student’s *t*-test ***P < 0.001. However, activation of BMP signaling pathway with constitutively active *tkv* receptors (*UAS-tkv^Q199D^* and *UAS-tkv^Q253D^*) led to strong lethality in in 386 > *shep-RNAi*, *Dcr-2*, *tub-Gal80^ts^* flies. EXW, expanded wings; PEW, partially expanded wings; UEW, unexpanded wings. (B and C) Expression of wild-type *tkv-EGFP* in 386 > *shep-RNAi*, *Dcr-2*, *tub-Gal80^ts^* flies fully rescued soma size of B_AG_ cells, but did not rescue soma sizes or neurite areas of B_SEG_ cells. Expression of constitutively active *tkv^Q253D^* receptors led to larger soma size and neurite areas of B_SEG_ cells. *P* < 0.001, One-way ANOVA [*** *P* < 0.001, Tukey’s honest significant difference (HSD) *post hoc*]. AG, abdominal ganglia; SEG, subesophageal ganglia. (D–F) Loss of *shep* led to loss of bursicon-positive neurites in the subesophageal and thoracic ganglia, and expression of constitutively active *tkv^Q253D^* receptors led to ectopic bursicon-positive neurites (arrows). Bar, 100 µm. (G and H) Expression of constitutively active *tkv^Q253D^* receptors led to ectopic bursicon-positive cells in the abdominal ganglia. Bar, 50 µm. (I) Quantification of bursicon-positive cell numbers in the abdominal ganglia. *P* < 0.001, one-way ANOVA (***P < 0.001, Tukey’s HSD *post hoc*).

As an alternative test of the model, we predicted that stimulation of the BMP signaling pathway would compensate for hyperactive *Dad* inhibition of BMP signaling and therefore phenocopy the suppression of loss-of-*shep* phenotypes by *Dad* RNAi or *Dad* mutant alleles. Flies carrying *UAS-tkv-EGFP*, which expresses wild-type *tkv* (Dudu *et al.* 2006), were crossed with 386 > *shep-RNAi*, *Dcr-2*, *tub-Gal80^ts^* flies at 30° to activate the BMP signaling pathway in loss-of-*shep* flies. The wing expansion defects were rescued by Gal4-directed expression of wild-type *tkv*; with *tkv*, 93% of the loss-of*-shep* progeny had fully expanded wings, while only 15% of the loss-of-*shep* progeny without *tkv* had fully expanded wings (Figure 7A). We performed anti-BURS immunostaining to visualize the bursicon neurons in these 386 > *shep-RNAi*, *Dcr-2*, *tkv-EGFP*, *tub-Gal80^ts^* animals, and we found that B_AG_ neuron soma size was fully restored by expression of *UAS-tkv-EGFP* (Figure 7B). Neurite projections and arbors of the B_AG_ or B_SEG_ were not rescued (Figure 7C). The rescue of B_AG_ neuron soma size and wing expansion, but not neurite projections or arbors, phenocopied the rescue of loss-of-*shep* by *Dad-RNAi* (Figure 5I), supporting our model that *shep* antagonizes *Dad* to regulate BMP signaling, and loss of *shep* led to hyperactive *Dad* inhibition of BMP signaling.

Because not all loss-of-*shep* phenotypes were rescued by *Dad* RNAi or wild-type *tkv*, we tested for suppression of the *shep* phenotypes after stronger activation of the BMP signaling pathway with *UAS-tkv^Q199D^* and *UAS-tkv^Q253D^*, which encode constitutively active *tkv* receptor alleles. When crossed to the 386 > *shep-RNAi*, *Dcr-2*, *tub-Gal80^ts^* flies at 30°, both strains carrying *UAS-tkv^Q199D^* or *UAS-tkv^Q253D^* produced late pupal lethality (Figure 7A), precluding a test for rescue of wing expansion defects. In both the brain and ventral nerve cord, we observed ectopic bursicon-positive neurites (Figure 7, C–F), and we also detected ectopic bursicon-positive cells in the ventral nerve cord (Figure 7, G–I). These ectopic neurites and cells indicated that hyperactive BMP signaling in the bursicon neurons and other peptidergic neurons in the *386-Gal4* pattern resulted in widespread changes in neuronal development and bursicon expression. To test effects of hyperactive BMP signaling alone on the bursicon neurons, we crossed *UAS-tkv-EGFP* or *UAS-tkv^Q253D^* into the *burs* > *Dcr-2* genetic background. While *burs* > *Dcr-2*, *tkv-EGFP* animals had normal wing expansion, *burs* > *Dcr-2*, *tkv^Q253D^* animals had 10% unexpanded wings and 7% partially expanded wings (*n* = 30), suggesting that hyperactive expression of *tkv* in the absence of *shep* RNAi caused abnormal development and/or function of bursicon neurons. Together with the results showing suppression by *Dad* RNAi and mutant alleles, these findings suggest that BMP signaling must be maintained within a defined window to support the normal development and/or function of bursicon neurons, and this balance is promoted by an antagonistic interaction between *shep* and the inhibitory Smad, *Dad*.

## Discussion

To identify *shep*-interacting factors and signaling pathways that contribute to the control of metamorphic neuronal remodeling, we screened 702 deficiencies located on the X, second, and third chromosomes for genetic modification of *shep* function in peptidergic neurons. These deficiencies together covered 86% of the euchromatic genes in the genome. We identified 24 suppressor deficiencies, and 12 of them rescued both the wing expansion and cellular defects of bursicon neurons caused by loss of *shep*. With RNAi, we mapped four individual suppressor loci: *CG10565*, *Myc*, *Oli*, and *Dad*. With mutant alleles, the suppression was further verified for *Oli* and *Dad*.

*Dad* encodes an inhibitory Smad protein in the BMP signaling pathway. Subsequent analysis revealed that activation of BMP signaling was sufficient to rescue both the wing expansion and cellular defects of bursicon neurons, and precisely regulated BMP signaling was essential for normal neuronal remodeling. Therefore, neuronal development in this context relies on precise regulation of BMP signaling that requires antagonism of *Dad* by *shep*.

### *shep* antagonizes *Dad* to regulate BMP signaling during neuronal remodeling

TGF-β ligands bind to type II membrane receptors, which recruit and phosphorylate type I receptors. The activated type I receptors then phosphorylate regulatory Smad proteins that dimerize with common Smad (co-Smad), and these complexes enter nuclei to function as transcription factors (Liu and Niswander 2005). The TGF-β signaling pathway is well conserved, and three type I receptors (Tkv, Sax, and Babo), two type II receptors (Punt and Wit), two R-Smads (Mad and dSmad2/Smox), and one Co-Smad (Medea) have been identified in *Drosophila* (Raftery *et al.* 2006; Kamiya *et al.* 2008). Signaling by two major classes of TGF-β superfamily ligands, BMP and activin, is divided into two different branches based on the partnering Smad proteins. The activin signaling pathway regulates neurite pruning during metamorphic neuronal remodeling, and disordered activin signaling leads to misexpressed EcR-B1 and interrupts neurite pruning in mushroom body neurons (Zheng *et al.* 2003; Awasaki *et al.* 2011; Yu *et al.* 2013) and motorneurons (Boulanger *et al.* 2012). BMP signaling promotes synaptic growth, regulates synaptic homeostasis in larval stages (Aberle *et al.* 2002; Marques *et al.* 2002; Berke *et al.* 2013; Sulkowski *et al.* 2014, 2016), and regulates neurite retraction of motorneurons during metamorphosis (Boulanger *et al.* 2012). To date, a role for BMP signaling in regulating the outgrowth phase of metamorphic remodeling has not been identified.

We isolated *Dad* as a strong suppressor of the wing expansion defects and cellular defects caused by loss of *shep*. Considering the inhibitory role of *Dad* in the BMP signaling pathway, we have proposed a model in which *shep* promotes BMP signaling by inhibiting *Dad* to ensure normal neuronal remodeling during metamorphosis. In support of this model, we showed that overexpression of wild-type *tkv* rescued the same loss-of-*shep* phenotypes that were rescued by *Dad* RNAi (Figure 5 and Figure 7, A–C). In wild-type *shep* animals, *Dad* RNAi did not promote overgrowth in the bursicon neurons, suggesting that the genetic interaction was not caused by nonspecific actions of *Dad* alone. In contrast, we observed ectopic bursicon-positive neurons and projections after expression of constitutively active forms of *tkv* (*e.g.*, *tkv^Q253D^*, Figure 7F), suggesting that hyperactive BMP signaling can induce bursicon expression in other cell types or regulate neurite development and bursicon expression in native bursicon neurons. Consistent with this model, SHEP interacts with *Dad* chromatin and negatively regulates *Dad* mRNA expression levels in neurons during metamorphic neuronal remodeling (personal communication, Elissa Lei, NIDDK, Bethesda, MD). Our model is also consistent with the interaction between the SHEP/MSSP proteins and TGF-β signaling characterized in craniofacial development of zebrafish (Jayasena and Bronner 2012), further suggesting that the interaction between SHEP/MSSP and TGF-β signaling may be evolutionarily conserved and shared across tissues. Together, these results suggest that *shep* negatively regulates *Dad* to maintain BMP signaling activity during neuronal remodeling.

We sometimes observed independent rescue of soma size or neurite morphology of bursicon neurons. For instance, *Dad* RNAi rescued both soma size and neurites (Figure 5, H and I), but the *Dad* deficiency (Figure 4 and Table S1 in File S2) and overexpression of *tkv* (Figure 7B) rescued only soma size. The partial rescue of cellular phenotypes may result from differential gene expression levels achieved in different mutant backgrounds. This is suggested by the differential effects of *Dad* RNAi and the *Dad* deficiency. However, it is also possible that different or overlapping mechanisms may control the growth of cell bodies and neurites. While some signaling pathways and factors are known to regulate neurite morphology (Williams and Truman 2005b; Kurtz *et al.* 2011; Yaniv *et al.* 2012; Gu *et al.* 2014; Medioni *et al.* 2014), the factors that shape neuron somata during neuronal remodeling remain undefined. If these mechanisms are different, then this system will provide an opportunity to understand distinct regulatory mechanisms controlling soma and neurite growth.

During *D. melanogaster* larval development, retrograde BMP signaling is essential for peptide expression in B_AG_ neurons (Veverytsa and Allan 2011, 2012). The loss of retrograde BMP signaling in these neurons leads to reduced expression of multiple neuropeptides and ecdysis defects (Veverytsa and Allan 2011). Reduced bursicon expression, ecdysis defects, and wing expansion defects are also observed in the cockroach *Blattella germanica* following reduction of TGF-β/BMP signaling (Santos *et al.* 2016), suggesting an evolutionarily conserved function of TGF-β/BMP in regulating neuropeptide expression. Therefore, the loss of *Dad* could also suppress the wing expansion defects seen in *shep* mutants by restoring (upregulating) bursicon expression. This seems unlikely, as the intensity of anti-BURS immunostaining in the bursicon neurons was strongly increased by *shep* RNAi (Figure 2E), but it remains possible given that the *shep* RNAi also reduces soma size, which could obscure any reduction in total bursicon expression by concentrating the remaining bursicon in a smaller area.

### Conserved antagonistic interaction between *shep* and *Myc*

We found that loss of *Myc* rescued the wing expansion and cellular defects of *shep*-depleted bursicon neurons. This is consistent with the known antagonism between the SHEP/MSSP family proteins and Myc in mouse fibroblasts. SHEP/MSSP proteins bind to the Myc/Max complex and inhibit its E-box based regulation of transcription (Niki *et al.* 2000b). Our findings explored this interaction in the process of neuronal development, and suggested that interaction between SHEP/MSSP and Myc was evolutionarily conserved. Myc protein binds to its own enhancer and may autoregulate its expression (Iguchi-Ariga *et al.* 1988), raising the possibility that SHEP antagonized Myc function by limiting Myc regulation of transcription, possibly the expression of Myc itself.

### Antagonistic interaction between *shep* and *Oli*

The vertebrate Olig family of basic Helix–Loop–Helix transcription factors have important functions during neuronal differentiation in multiple systems (Lee and Pfaff 2003; Lee *et al.* 2004; Joshi *et al.* 2008; Ross *et al.* 2010, 2012). Our results show that *Oli* also interacts with *shep* to regulate the development of peptidergic neurons. Interestingly, zygotic SHEP is not detected until late embryonic stage 17 (Chen *et al.* 2014) when *Oli* expression is downregulated (Graveley *et al.* 2011; Oyallon *et al.* 2012). Based on this negative correlation and our finding that *shep* and *Oli* have opposing functions during metamorphic remodeling of bursicon neurons, it will be important to determine whether either SHEP or Oli inhibits the expression or function of the other.

In summary, we identified novel genetic interactions between *shep* and 24 genomic suppressor loci, from which we were able to map four individual suppressors, *Dad*, *Myc*, *Oli*, and *CG10565*, that interact with *shep* to regulate neuronal remodeling during metamorphosis. Our results further suggest that BMP signaling is regulated by *shep* antagonism of *Dad* to regulate neuronal remodeling during metamorphosis. We also provided evidence of a genetic interaction between *shep* and *su*(*Hw*) to regulate wing expansion. These findings illustrate the biological significance of known SHEP/MSSP interactions with *Myc* or chromatin insulators in the context of metamorphic neuronal remodeling, and they also reveal novel interactions between *shep* and the BMP signaling pathway to regulate neuronal remodeling during metamorphosis.

## Supplementary Material

Supplemental material is available online at www.genetics.org/lookup/suppl/doi:10.1534/genetics.117.200378/-/DC1.
