# Supplementary material for: Regulatory Mechanisms of Metamorphic Neuronal Remodeling Revealed Through a Genome-Wide Modifier Screen in Drosophila melanogaster
Source: Genetics. 2017 May 5;206(3):1429–43. doi: 10.1534/genetics.117.200378 (PMC5500141; doi:10.1534/genetics.117.200378)
Supplement: Supplementary file 1 [file 1429FigureS1.pdf]

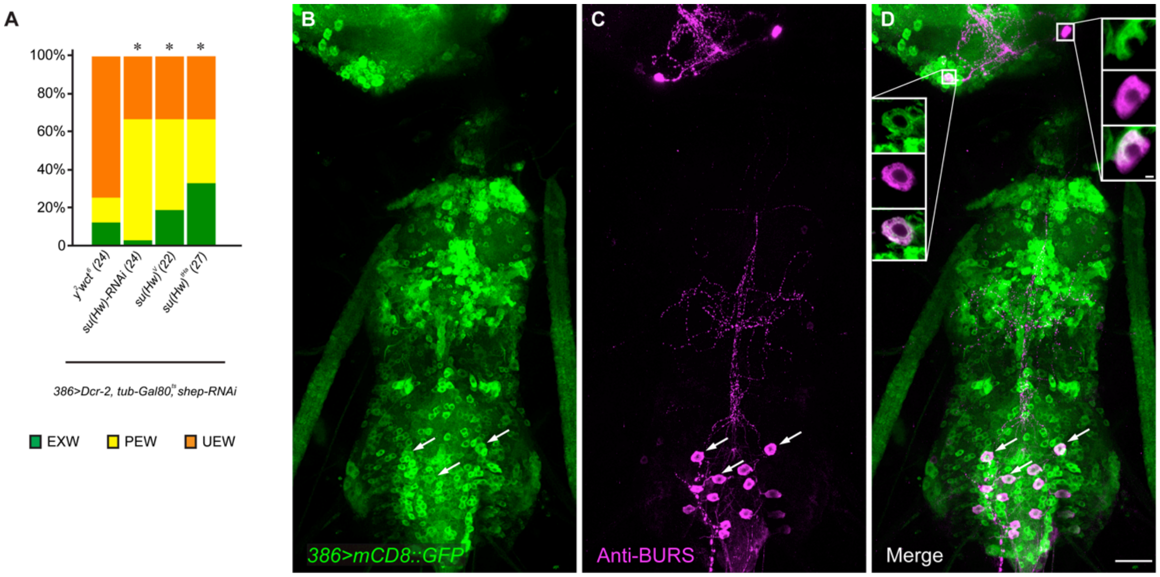

Figure S1. The bursicon neurons were a subgroup of neurons in the *386-Gal4* pattern. (A) Suppression of the wing expansion defects by the *su(Hw)* gene. The mutant alleles, *su(Hw)*^{V} with or without *su(Hw)*^{tHa}, and *su(Hw)* RNAi suppressed the wing expansion defects when crossed to the *386>shep-RNAi, Dcr-2, tub-Gal80*^{ts} test stock. (*) *P*<0.05, (**) *P*<0.01, (***) *P*<0.001, Fisher's exact test with Bonferroni correction. Sample sizes in parentheses. (B) *386-Gal4* was used to express *UAS-mCD8::GFP* in a P14 stage pharate adult CNS. (C) The same nervous system was labeled by anti-BURS immunostaining. (D) Merged image. Both the B_{SEG} (insets) and B_{AG} neurons (arrows) displayed co-localization of the mCD8::GFP reporter and BURS. Scale bars: 50 µm; insets, 3 µm.
