# Supplementary material for: Regulatory Mechanisms of Metamorphic Neuronal Remodeling Revealed Through a Genome-Wide Modifier Screen in Drosophila melanogaster
Source: Genetics. 2017 May 5;206(3):1429–43. doi: 10.1534/genetics.117.200378 (PMC5500141; doi:10.1534/genetics.117.200378)
Supplement: Supplementary file 2 [file 1429FigureS2.pdf]

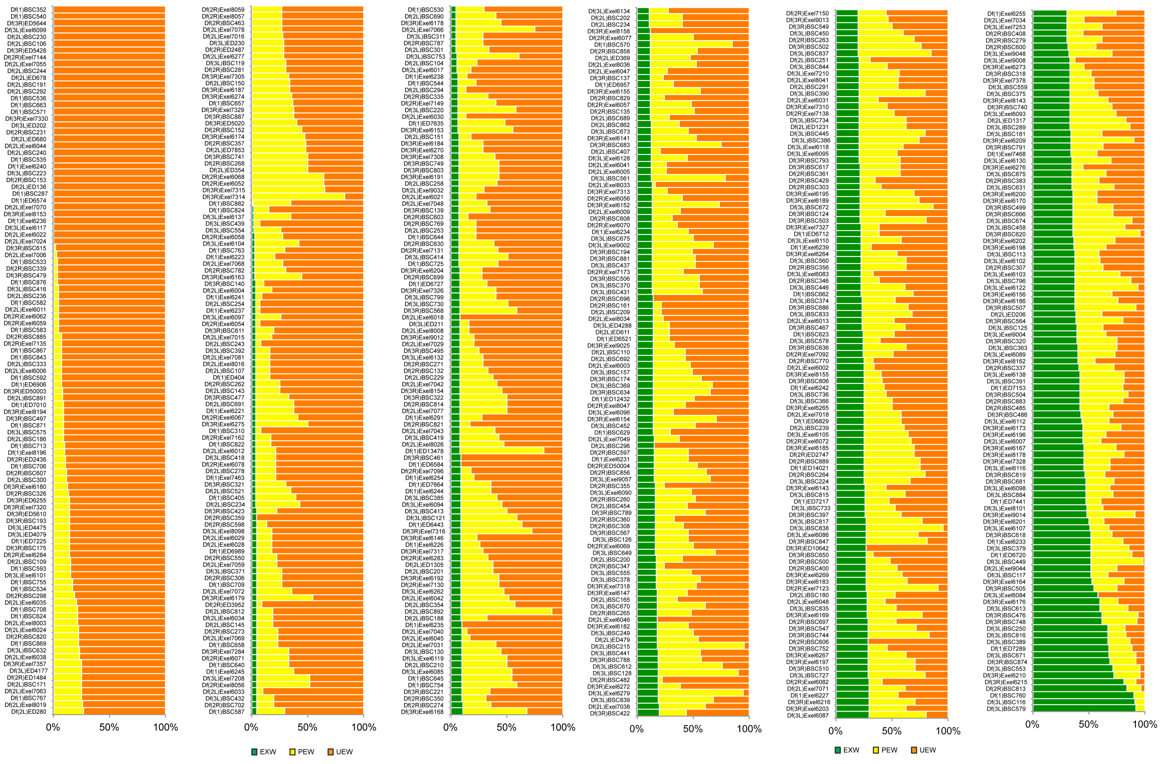

Figure S2. Wing expansion percentages for test crosses with deficiencies. Wing expansion rates were quantified and plotted for progeny of test crosses between *386>shep-RNAi, Dcr-2, tub-Gal80ts* flies and the 633 deficiencies that generated adult progeny. The deficiencies were sorted for this figure based on the EXW and then the PEW scores.
