# Supplementary material for: Regulatory Mechanisms of Metamorphic Neuronal Remodeling Revealed Through a Genome-Wide Modifier Screen in Drosophila melanogaster
Source: Genetics. 2017 May 5;206(3):1429–43. doi: 10.1534/genetics.117.200378 (PMC5500141; doi:10.1534/genetics.117.200378)
Supplement: Supplementary file 3 [file 1429FigureS3.pdf]

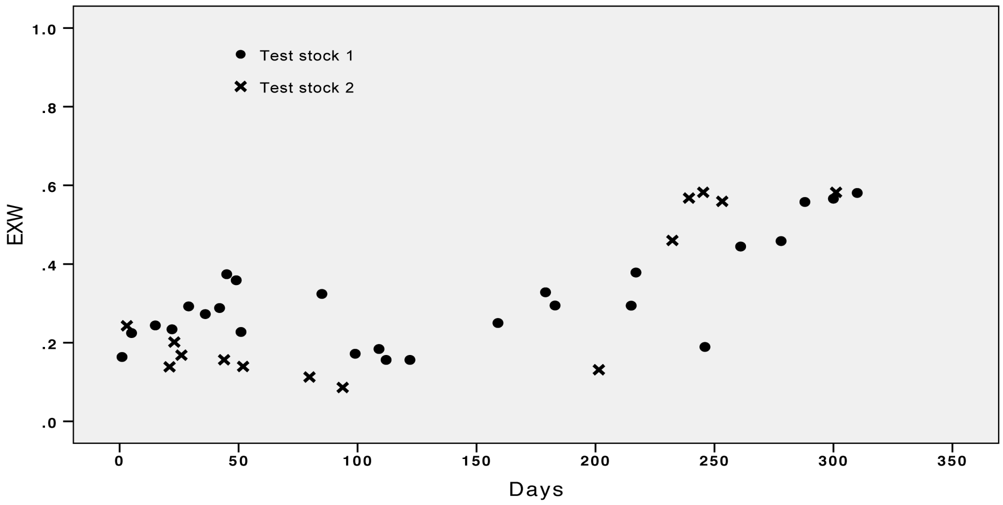

Figure S3. Phenotypic drift of control crosses with the *60100* genetic background strain. Wing expansion rates in control crosses with two separately prepared test stocks, *386>shep-RNAi, Dcr-2, tub-Gal80^{ts}*. The percentage of EXW adults obtained with the test stock 1 was plotted as a function of time with filled circles, and the scores obtained with test stock 2 were plotted with crosses. The day a test stock was used for the first time was set as day 1.
