# Supplementary material for: Regulatory Mechanisms of Metamorphic Neuronal Remodeling Revealed Through a Genome-Wide Modifier Screen in Drosophila melanogaster
Source: Genetics. 2017 May 5;206(3):1429–43. doi: 10.1534/genetics.117.200378 (PMC5500141; doi:10.1534/genetics.117.200378)
Supplement: Supplementary file 4 [file 1429FigureS4.pdf]

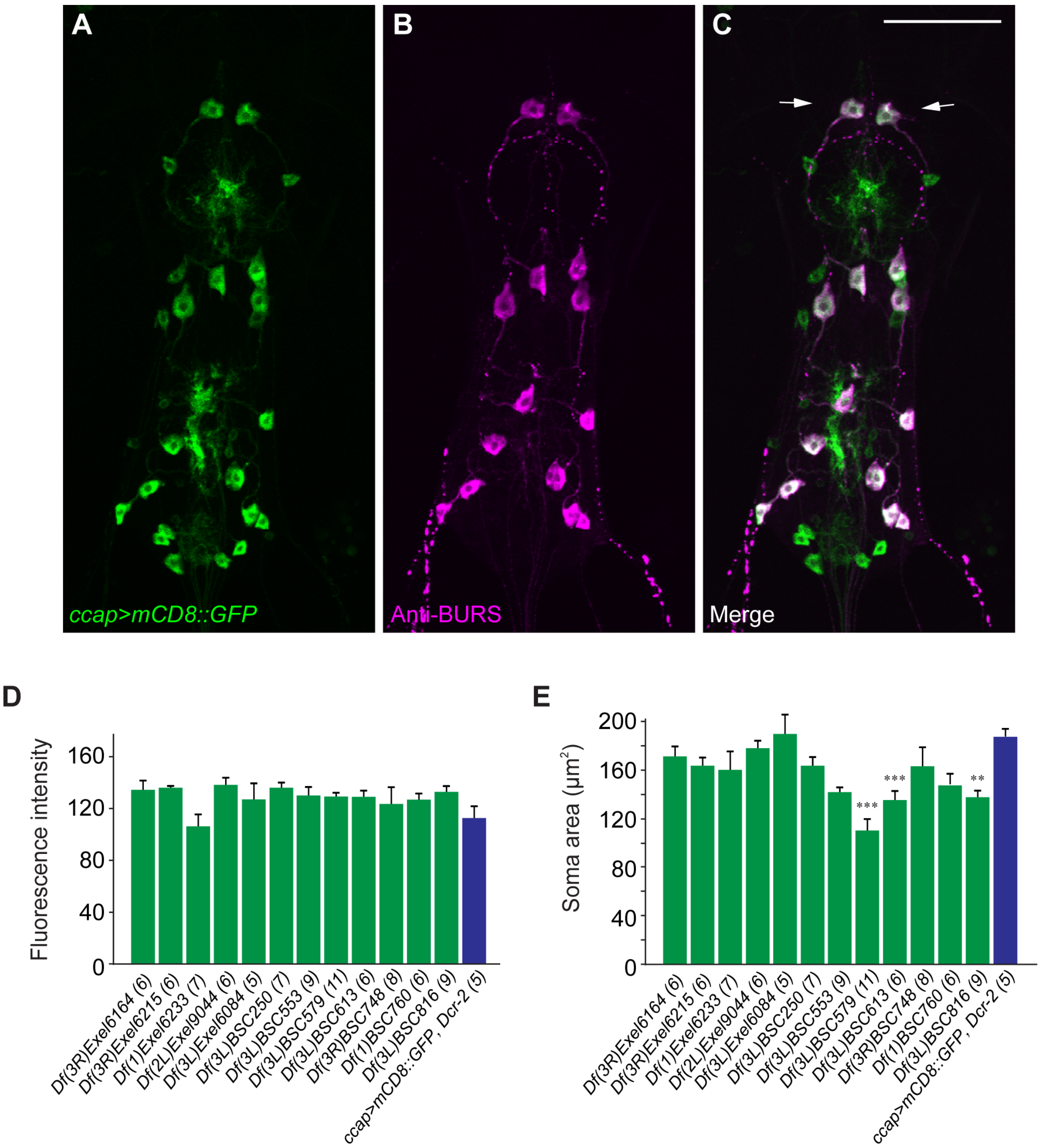
A
B
C
ccap>mCD8::GFP
Anti-BURS
Merge
D
Fluorescence intensity
0
40
80
120
160
E
Soma area (μm²)
0
40
80
120
160
200
Df(3R)Exel6164 (6)
Df(3R)Exel6215 (6)
Df(1)Exel6233 (7)
Df(2L)Exel9044 (6)
Df(3L)Exel6084 (5)
Df(3L)BSC250 (7)
Df(3L)BSC553 (9)
Df(3L)BSC579 (11)
Df(3L)BSC613 (6)
Df(3R)BSC748 (8)
Df(1)BSC760 (6)
Df(3L)BSC816 (9)
ccap>mCD8::GFP, Dcr-2 (5)
***
***
**

Figure S4. Suppressor deficiencies did not directly promote growth or affect expression of an mCD8::GFP reporter. (A-C) The B_{AG} neurons expressed bursicon peptides (panel B, anti-BURS immunostaining) that were co-localized with mCD8::GFP driven under control of the *ccap-Gal4* driver (panel A). Panel C shows the merged image. Scale bar: 100 μm. (D) Expression levels of mCD8::GFP in the most anterior pair of B_{AG} neurons (arrows) of *ccap>mCD8::GFP/deficiency* animals, measured as soma fluorescence. P>0.931, One-way ANOVA. (E) B_{AG} neuron soma areas resulting from the same crosses as in panel D. The most anterior pair of B_{AG} neurons (arrowheads in panel C) was examined. None of the suppressor deficiencies promoted cell growth, although three of them significantly reduced soma size. P<0.000001, One-way ANOVA (***, P<0.001, **, P<0.01, Tukey HSD *post hoc*).
