# Supplementary material for: Regulatory Mechanisms of Metamorphic Neuronal Remodeling Revealed Through a Genome-Wide Modifier Screen in Drosophila melanogaster
Source: Genetics. 2017 May 5;206(3):1429–43. doi: 10.1534/genetics.117.200378 (PMC5500141; doi:10.1534/genetics.117.200378)
Supplement: Supplementary file 5 [file 1429FigureS5.pdf]

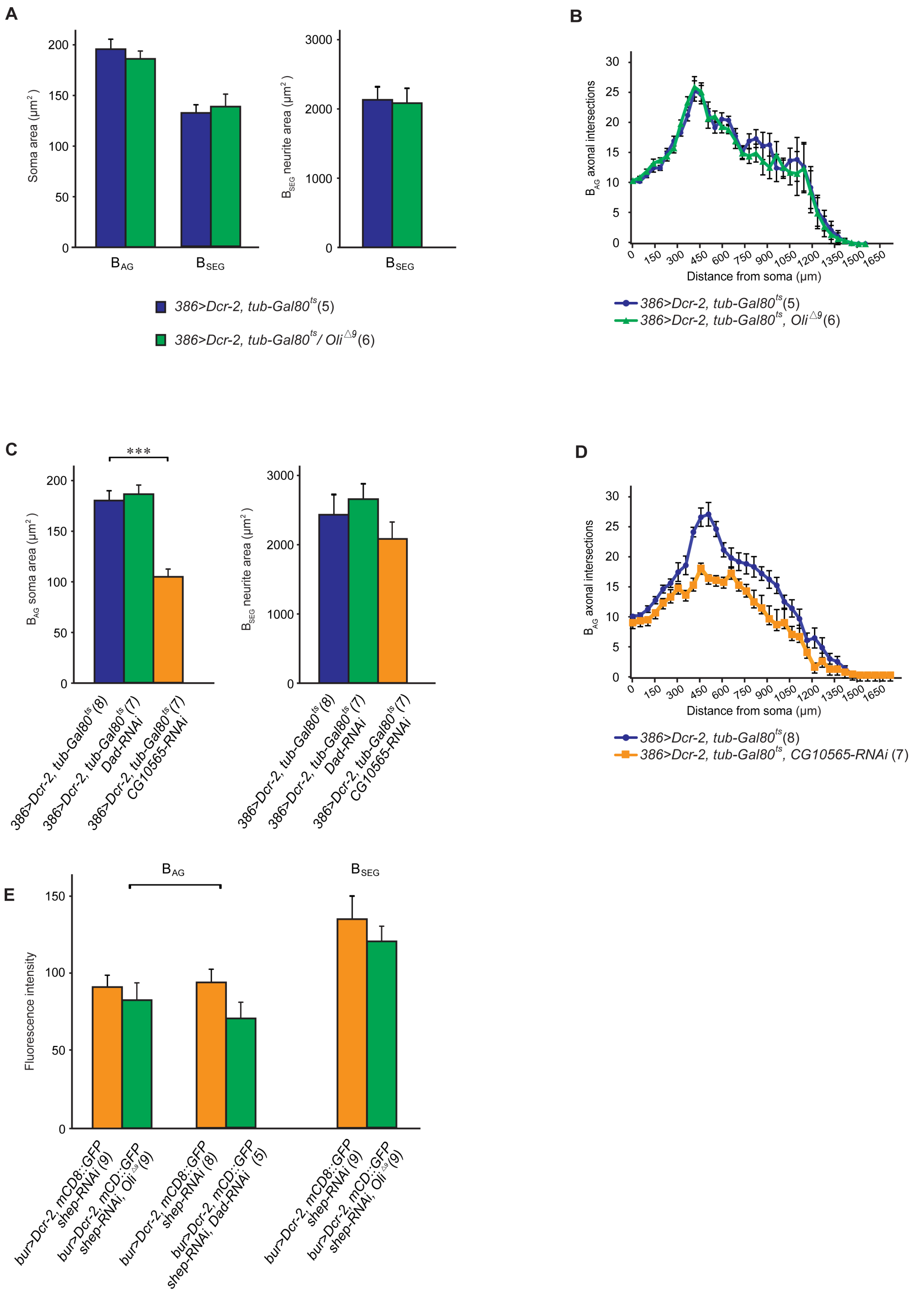
A
Soma area (µm²)
B_AG
B_SEG
B_SEG neurite area (µm²)
386>Dcr-2, tub-Gal80ts(5)
386>Dcr-2, tub-Gal80ts/ Oli△9(6)
B
B_AG axonal intersections
Distance from soma (µm)
386>Dcr-2, tub-Gal80ts(5)
386>Dcr-2, tub-Gal80ts, Oli△9(6)
C
***
B_AG soma area (µm²)
B_SEG neurite area (µm²)
386>Dcr-2, tub-Gal80ts (8)
386>Dcr-2, tub-Gal80ts (7) Dad-RNAi
386>Dcr-2, tub-Gal80ts (7) CG10565-RNAi
D
B_AG axonal intersections
Distance from soma (µm)
386>Dcr-2, tub-Gal80ts(8)
386>Dcr-2, tub-Gal80ts, CG10565-RNAi (7)
E
B_AG
B_SEG
Fluorescence intensity
bur>Dcr-2, mCD8::GFP shep-RNAi (9)
bur>Dcr-2, mCD::GFP shep-RNAi, Oli△9 (9)
bur>Dcr-2, mCD8::GFP shep-RNAi (8)
bur>Dcr-2, mCD::GFP shep-RNAi, Dad-RNAi (5)
bur>Dcr-2, mCD8::GFP shep-RNAi (9)
bur>Dcr-2, mCD::GFP shep-RNAi, Oli△9 (9)

Figure S5. RNAi or mutant allele transgenes did not affect morphology of bursicon neurons on their own. (A-B) The *Oli*$^{\Delta 9}$ mutant allele did not affect soma size or neurite morphology (B, Sholl analysis) of the bursicon neurons in the absence of *shep* RNAi. P>0.05, Student's t-test. (C) *Dad* RNAi did not affect morphology of bursicon neurons in the absence of *shep* RNAi. *CG10565* RNAi alone was sufficient to result in smaller B_{AG} soma sizes. P< 0.00001, One-way ANOVA, (***, P<0.001, Tukey HSD *post hoc*). (D) *CG10565* RNAi alone was sufficient to result in fewer axonal projections of B_{AG} cells. (E) Both *Oli*$^{\Delta 9}$ and *Dad* RNAi showed similar GFP expression levels in the B_{AG} and B_{SEG} cells, suggesting that they suppressed loss-of-*shep* phenotypes without affecting general transcription levels. P>0.05, Student's t-test.
