# Supplementary material for: Regulatory Mechanisms of Metamorphic Neuronal Remodeling Revealed Through a Genome-Wide Modifier Screen in Drosophila melanogaster
Source: Genetics. 2017 May 5;206(3):1429–43. doi: 10.1534/genetics.117.200378 (PMC5500141; doi:10.1534/genetics.117.200378)
Supplement: Supplementary file 6 [file 1429FigureS6.pdf]

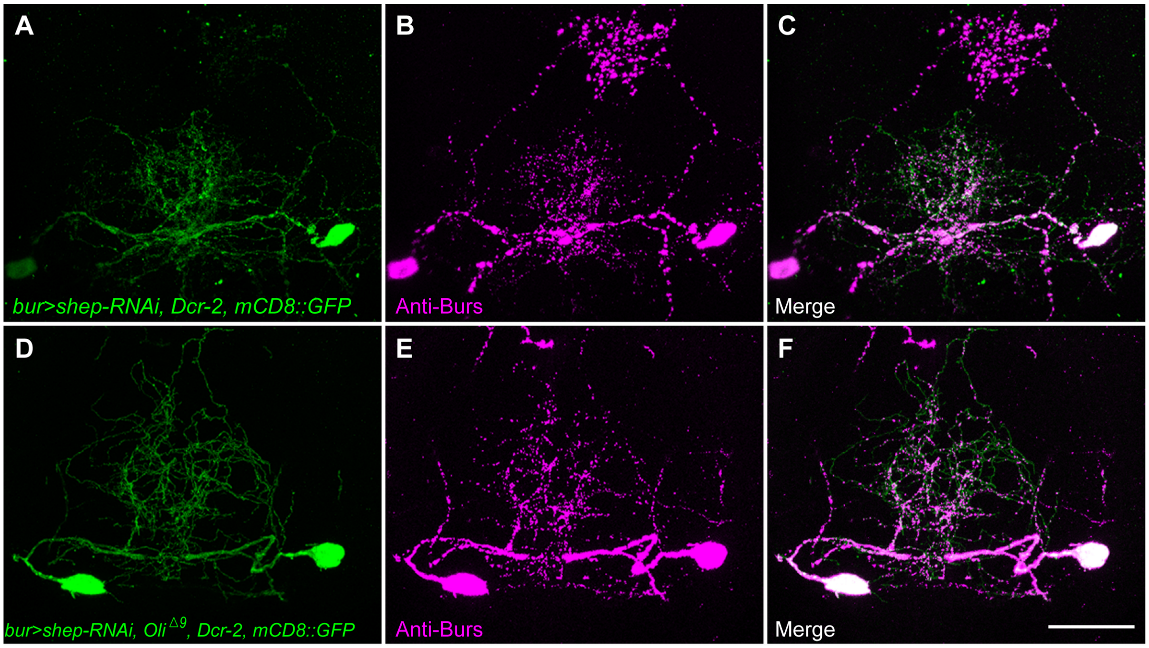

Figure S6. The neurite arbor of the B_{SEG} neurons was resolved with anti-BURS immunostaining and mCD8::GFP. (A-C) Anti-BURS immunostaining (panel A) and the mCD8::GFP membrane tag (panel B) provided similar resolution of the B_{SEG} neurites (panel C). (D-F) The equivalent labeling by anti-BURS (panel D) and mCD8::GFP (panel E) was maintained in loss-of-*shep* animals with a heterozygous *Oli*$^{\Delta 9}$ allele (panel F, merge).
