# Supplementary material for: Regulatory Mechanisms of Metamorphic Neuronal Remodeling Revealed Through a Genome-Wide Modifier Screen in Drosophila melanogaster
Source: Genetics. 2017 May 5;206(3):1429–43. doi: 10.1534/genetics.117.200378 (PMC5500141; doi:10.1534/genetics.117.200378)
Supplement: Supplementary file 8 [file 1429FileS2.docx]

**Table S1.** Progeny lethality of crosses with the test stock and deficiencies

| Phenotype(s) | Deficiencies |
| --- | --- |
| Lethality before pupariation | *Df(1)ED7229, Df(1)ED7620, Df(2L)ED5878, Df(2L)ED19, Df(2L)ED6569, Df(2L)ED779, Df(2L)BSC768, Df(2R)Exel6064, Df(3L)ED224, Df(3R)ED5518, Df(1)BSC705, Df(2R)Exel6055, Df(3L)BSC443, Df(3L)ED220, Df(3R)ED5177, Df(3R)ED10893, Df(3L)ED225, Df(3L)ED4786, Df(3R)BSC633, Df(1)ED411, Df(1)BSC710, Df(2L)Exel6036, Df(2L)Exel6039, Df(3L)ED4293, Df(3R)ED6235, Df(1)BSC580, Df(2R)BSC639, Df(3R)BSC808, Df(2R)BSC880, Df(2L)ED1054, Df(3L)ED4529, Df(3R)ED5591,Df(3R)ED10845, Df(3R)ED6316, Df(3R)ED6361,* |
| Head eversion defect | *Df(3R)Exel6158, Df(2R)Exel6076* |
| Head eversion defect and late pupal lethality | *Df(3R)Exel6145, Df(3R)Exel6188, Df(3L)Exel6127, Df(3L)BSC800, Df(3R)ED6058, Df(3R)ED6346* |
| Late pupal lethality | *Df(2L)ED108, Df(2R)ED3923, Df(3L)ED4502, Df(3L)ED4543, Df(3R)ED6103, Df(3L)ED5017, Df(3R)ED5021, Df(3L)ED217, Df(3L)Exel6131, Df(3L)Exel6092, Df(3R)ED5147, Df(3R)ED5223, Df(3L)Exel6109, Df(3L)ED4789, Df(3L)ED4799, Df(3L)BSC797, Df(3R)Exel6181, Df(2R)E.D3791, Df(3L)ED4414, Df(3L)ED4457, Df(3L)ED4978, Df(3R)ED5156, Df(3R)Exel6214,* |
| Late pupal lethality and eclosion defect | *Df(2L)Exel8038, Df(2R)ED2098* |
| Eclosion defect | *Df(3L)Exel6123* |

**Table S2.** The 24 top suppressor deficiencies obtained in the *shep* modifier screen.

| Deficiency | Flybase ID | | Overlapping/nested deficiencies tested | B_AG_ rescue | B_SEG_ rescue | Deleted or immediately flanking protein-encoding gene(s)^a^ |
| --- | --- | --- | --- | --- | --- | --- |
| *Df(3R)Exel6164* | FBst0007643 | | *Df(3R)Exel7315, Df(3R)Exel7316* | Y | Y | ***MBD*, *svp*, *CG10013*, *CG10038*, *CG10041*, *CG4115*, *Tim17a1*, *CG18548***, *CG10091*, *CG4066, GstD9, GstD1, GstD10* |
| *Df(1)Exel6233* | FBst0007707 | | *Df(1)Exel6712* | N | Y | *M****yc*** |
| *Df(2L)Exel9044* | FBst0007836 | | N/A | Y | Y | ***CG6412*, *Oli*, *CG6870*, *syntalpha*, *CG15143*, *CG15144*, *CG15145*, *CG7094***, *Dhc* |
| *Df(3L)BSC250* | FBst0023150 | | *Df(3L)BSC363, Df(3L)BSC431, Df(3L)BSC311, Df(3L)BSC289* | Y | Y | ***CG9149*, *CG2277*, *CG2469*, *CG9186*, *CG9153*, *Myo61F*, *CG9184*, *mtacp1***, *msd1* |
| *Df(3L)BSC553* | FBst0025116 | | *Df(3L)BSC449* | Y | N | ***Sin*, *siz*, *CG12984*, *CG10584*, *CG10585*, *CG10581*, *CG33284*, *CG10566*, *CG10565***, *CG43072*, *CG43938*, *ko*, *skd*, *CG33285*, *Ac78C, asparagine-synthetase* |
| *Df(3L)BSC613* | FBst0025688 | | *Df(3L)Exel6117* | Y | Y | ***CG10948*, *CG42709*, *CG32110*, *CG42588***, *CG10973*, *CG32113*, *Hip1*, *CG32106*, *CG10969*, *CG17666*, *Atg1*, *Sap130*, *CG10754*, *CG10960* |
| *Df(3L)BSC816* | FBst0027577 | | *Df(3L)BSC612,* *Df(3L)ED4110* | Y | Y | ***Cpr66D*, *pex7*, *CG13305*, *Zasp66***, *Arr2, hairy* |
| *Df(3R)BSC748* | FBst0026846 | | *Df(3R)Exel6176* | Y | N | ***Dad*** |
| *Df(3L)Exel6084* | FBst0007563 | | *Df(3L)BSC121, Df(3L)BSC128, Df(3L)BSC125, Df(3L)BSC126, Df(3L)ED4177* | Y | N | ***Kaz1*, *DIP2***, *pyx*, *E(bx)*, *mthl14*, *CG13877*, *CG33229*, *CG34453*, *CG42846*, *thoc7*, *CG34454*, *CG16940*, *CG34263*, *mRpL17*, *Tudor*-*SN*, *wac*, *miple*, *miple2*, *CG32845*, *CG7028* |
| *Df(3L)BSC579* | FBst0025413 | | *Df(3L)BSC559, Df(3L)BSC560, Df(3L)BSC649* | Y | Y | *Nplp3*, *CG13041*, *CG13060*, *CG42718*, *CG13059*, *CG13062* |
| *Df(3R)Exel6215* | FBst0007693 | | *Df(3R)BSC503, Df(3R)BSC504, Df(3R)Exel6216, Df(3R)Exel7378* | Y | Y | *Tmod*, *CG34155* |
| *Df(1)BSC760* | FBst0026857 | | N/A | Y | Y | *Para*, *CG9903*, *CG9902*, *Arp2*, *Pp28-14D*, *CanA-14F*, *CG13014*, *Cap*, *UBc7, CG9784*, *Nup153*, *mbt*, *CG9782*, *rok*, *CG9777*, *Cnx14D* |
| *Df(3R)Exel6210* | FBst0007688 | | *Df(3R)BSC567, Df(3R)BSC789, Df(3R)BSC806, Df(3R)BSC322, Df(3R)Exel6209, Df(3R)BSC874* | N | N | *CG1646*, *wdn*, *CG1523*, *CG1647*, *CheB98a* |
| *Df(3L)BSC116* | FBst0008973 | | *Df(3L)BSC385, Df(3L)Exel6090* | N | N | *CG1317, CG8993, mRpL23*, *CG9004, CG15877, CG45186, CG42787, CG32298, CG32299,* |
| *Df(3L)BSC389* | FBst0024413 | | *Df(3L)BSC157* | N/A | N/A | *ergic53*, *rhea*, *CG6638*, *CG43078* |
| *Df(3R)BSC476* | FBst0024980 | | *Df(3R)Exel6264, Df(3R)Exel6153* | N | N | *Alpha-Man-II, ps*, *GstZ1*, *GstZ2*, *CG16779*, *CG8147*, *RhoL*, *CG8149*, *rump*, *Ras85D, Rlb1*, *mRpL47* |
| *Df(1)ED7289* | FBst0029732 | | *Df(1)BSC310* | N | N | *CG5599*, *Rab3-GEF*, *CG9072*, *Cyp4s3*, *drd* |
| *Df(1)ED6720* | FBst0009055 | Eliminated since overlapping or nested deficiencies that covered the entire candidate region were tested  yet failed to display suppression. | | | | |
| *Df(3L)BSC671* | FBst0026523 |  |  |  |  |  |
| *Df(2R)BSC813* | FBst0027384 |  |  |  |  |  |
| *Df(3R)BSC505* | FBst0025009 | Eliminated due to its potential effect on RNAi efficacy by deleting *pasha* | | | | |

The column labeled “B_AG_ rescue” indicates whether the B_AG_ soma size defects caused by *shep* RNAi were partially or completely suppressed. The “B_SEG_ rescue” column indicates whether B_SEG_ arbor in the ventral nerve cord was partially or completely restored. Genes partially or completely deleted by each deficiency, together with flanking genes located within 3 kb of the deficiency breakpoints (underlined), are listed.

^a^Bold, genes tested by RNAi.

**Table S3.** Progeny lethality produced by crosses with the tester strain to RNAi strains

| Phenotype(s) | Targets of the VDRC RNAi strains |
| --- | --- |
| Head eversion defect and late pupal lethality | *CG10042* |
| Late pupal lethality | *CG42588*, *CG6412*, *CG10566*, *CG33284*, *mtacp1*, *Sin* |
