# Supplementary material for: Regulatory Mechanisms of Metamorphic Neuronal Remodeling Revealed Through a Genome-Wide Modifier Screen in Drosophila melanogaster
Source: Genetics. 2017 May 5;206(3):1429–43. doi: 10.1534/genetics.117.200378 (PMC5500141; doi:10.1534/genetics.117.200378)
Supplement: Supplementary file 9 [file 1429FileS3.docx]

**Fig S1. The bursicon neurons were a subgroup of neurons in the *386-Gal4* pattern.** (A) Suppression of the wing expansion defects by the *su(Hw)* gene. The mutant alleles, *su(Hw)^V^* with or without *su(Hw)^tHa^*, and *su(Hw)* RNAi suppressed the wing expansion defects when crossed to the *386>shep-RNAi, Dcr-2, tub-Gal80^ts^* test stock. (*) *P*<0.05, (**) *P*<0.01, (***) *P*<0.001, Fisher’s exact test with Bonferroni correction. Sample sizes in parentheses. (B) *386-Gal4* was used to express *UAS-mCD8::GFP* in a P14 stage pharate adult CNS. (C) The same nervous system was labeled by anti-BURS immunostaining. (D) Merged image. Both the B_SEG_ (insets) and B_AG_ neurons (arrows) displayed co-localization of the mCD8::GFP reporter and BURS. Scale bars: 50 µm; insets, 3 µm.

**Fig S2. Wing expansion percentages for test crosses with deficiencies.**  Wing expansion rates were quantified and plotted for progeny of test crosses between *386>shep-RNAi, Dcr-2, tub-Gal80ts* flies and the 633 deficiencies that generated adult progeny. The deficiencies were sorted for this figure based on the EXW and then the PEW scores.

**Fig S3. Phenotypic drift of control crosses with the *60100* genetic background strain.** Wing expansion rates in control crosses with two separately prepared test stocks, *386>shep-RNAi, Dcr-2, tub-Gal80^ts^*. The percentage of EXW adults obtained with the test stock 1 was plotted as a function of time with filled circles, and the scores obtained with test stock 2 were plotted with crosses. The day a test stock was used for the first time was set as day 1.

**Fig S4. Suppressor deficiencies did not directly promote growth or affect expression of an mCD8::GFP reporter.** (A-C) The B_AG_ neurons expressed bursicon peptides (panel B, anti-BURS immunostaining) that were co-localized with mCD8::GFP driven under control of the *ccap-Gal4* driver (panel A). Panel C shows the merged image. Scale bar: 100 µm. (D) Expression levels of mCD8::GFP in the most anterior pair of B_AG_ neurons (arrows) of *ccap>mCD8::GFP/deficiency* animals, measured as soma fluorescence. *P*>0.931, One-way ANOVA. (E) B_AG_ neuron soma areas resulting from the same crosses as in panel D. The most anterior pair of B_AG_ neurons (arrowheads in panel C) was examined. None of the suppressor deficiencies promoted cell growth, although three of them significantly reduced soma size. *P*<0.000001, One-way ANOVA (***, *P*<0.001, **, *P*<0.01, Tukey HSD *post hoc*).

**Fig S5. RNAi or mutant allele transgenes did not affect morphology of bursicon neurons on their own.** (A-B) The *Oli^Δ9^* mutant allele did not affect soma size or neurite morphology (B, Sholl analysis) of the bursicon neurons in the absence of *shep* RNAi. *P*>0.05, Student’s *t*-test. (C) *Dad* RNAi did not affect morphology of bursicon neurons in the absence of *shep* RNAi. *CG10565* RNAi alone was sufficient to result in smaller B_AG_ soma sizes. *P*< 0.00001, One-way ANOVA, (***, *P*<0.001, Tukey HSD *post hoc*). (D) *CG10565* RNAi alone was sufficient to result in fewer axonal projections of B_AG_ cells. (E) Both *Oli^Δ9^* and *Dad* RNAi showed similar GFP expression levels in the B_AG_ and B_SEG_ cells, suggesting that they suppressed loss-of-*shep* phenotypes without affecting general transcription levels. *P*>0.05, Student’s *t*-test.

**Fig S6. The neurite arbor of the B_SEG_ neurons was resolved with anti-BURS immunostaining and mCD8::GFP.**  (A-C) Anti-BURS immunostaining (panel A) and the mCD8::GFP membrane tag (panel B) provided similar resolution of the B_SEG_ neurites (panel C). (D-F) The equivalent labeling by anti-BURS (panel D) and mCD8::GFP (panel E) was maintained in loss-of-*shep* animals with a heterozygous *Oli^Δ9^* allele (panel F, merge).

**File S1. 3D structure of the B_SEG_ neurite arbor in the brain and subesophageal ganglia.**  The animation is a tracing of anti-bursicon immunostaining in a P14 pharate adult brain.

**Table S1.** Progeny lethality of crosses with the test stock and deficiencies.

**Table S2.** The 24 top suppressor deficiencies obtained in the *shep* modifier screen.

**Table S3.** Progeny lethality produced by crosses with the tester strain to RNAi strains.
